# Core-predominant gut fungus *Kazachstania slooffiae* promotes intestinal epithelial glycolysis via lysine desuccinylation in pigs

**DOI:** 10.1186/s40168-023-01468-3

**Published:** 2023-02-23

**Authors:** Jun Hu, Jianwei Chen, Qiliang Hou, Xiaojian Xu, Jing Ren, Libao Ma, Xianghua Yan

**Affiliations:** 1 State Key Laboratory of Agricultural Microbiology, Hubei Hongshan Laboratory, Frontiers Science Center for Animal Breeding and Sustainable Production, College of Animal Sciences and Technology, Huazhong Agricultural University, Wuhan, 430070 Hubei China; 2 The Cooperative Innovation Center for Sustainable Pig Production, Wuhan, 430070 Hubei China; 3 Hubei Provincial Engineering Laboratory for Pig Precision Feeding and Feed Safety Technology, Wuhan, 430070 Hubei China; 4 BGI Research-Qingdao, BGI, Qingdao, 266555 China

**Keywords:** Gut fungi, *Kazachstania slooffiae*, Lysine succinylation, Intestinal epithelium, Glycolysis, Sirtuin 5

## Abstract

**Background:**

Gut fungi are increasingly recognized as important contributors to host physiology, although most studies have focused on gut bacteria. Post-translational modifications (PTMs) of proteins play vital roles in cell metabolism. However, the contribution of gut fungi to host protein PTMs remains unclear. Mining gut fungi that mediate host protein PTMs and dissecting their mechanism are urgently needed.

**Results:**

We studied the gut fungal communities of 56 weaned piglets and 56 finishing pigs from seven pig breeds using internal transcribed spacer (ITS) gene amplicon sequencing and metagenomics. The results showed that *Kazachstania slooffiae* was the most abundant gut fungal species in the seven breeds of weaned piglets. *K. slooffiae* decreased intestinal epithelial lysine succinylation levels, and these proteins were especially enriched in the glycolysis pathway. We demonstrated that *K. slooffiae* promoted intestinal epithelial glycolysis by decreasing lysine succinylation by activating sirtuin 5 (SIRT5). Furthermore, *K. slooffiae*-derived 5′-methylthioadenosine metabolite promoted the SIRT5 activity.

**Conclusions:**

These findings provide a landscape of gut fungal communities of pigs and suggest that *K. slooffiae* plays a crucial role in intestinal glycolysis metabolism through lysine desuccinylation. Our data also suggest a potential protective strategy for pigs with an insufficient intestinal energy supply.

Video Abstract

**Supplementary Information:**

The online version contains supplementary material available at 10.1186/s40168-023-01468-3.

## Introduction

Growing evidence has revealed that gut bacteria play important roles in host health [1]. The volume of a typical fungal cell is about more than 100-fold that of a typical bacterial cell, suggesting a much more substantial mass of biomaterials [2]. The intestinal tract harbors a variety of fungal species [3–6] and these gut fungi have the potential to exhibit unique metabolic features [2, 7]. Gut fungal communities are also disordered in patients with various diseases, including colorectal cancer (CRC) [8], primary sclerosing cholangitis [9], and inflammatory bowel disease (IBD) [10]. Recent studies have reported that gut fungi also play important roles in regulating host metabolism and immunity [2, 11–15]. The intestinal tract harbors core microbes that are predominant in the gut microbiota, suggesting a stable symbiosis between the host and core gut microbes [16, 17]. Thus, core-predominant gut fungal species in the gut may have powerful metabolic activities in the intestinal tract and critical regulatory roles in the host metabolism.

Post-translational modifications (PTMs) are critical steps in protein maturation and have important effects on cell metabolism, innate immunity, and pathogenesis [18]. Interestingly, the protein expression [19, 20], as well as protein PTMs, such as phosphorylation and crotonylation [21–23] in host cells, can be mediated by microbes, including viruses and bacteria. Many bacterial virulence effector proteins have been demonstrated to play important roles in protein PTMs, such as ubiquitination, glycosylation, fatty acylation, and ADP-ribosylation, in modulating host signal transduction [24–28]. However, it is unclear whether gut fungi induce alterations in host protein PTMs, and the underlying mechanisms have not yet been elucidated. Thus, investigating the gut fungi-induced alterations in host protein PTMs is of great significance and contributes to providing new perspectives on the mechanism of host–gut fungi interactions.

In this study, we analyzed the landscape of fungal communities in fecal samples from 56 weaned piglets and 56 finishing pigs from seven pig breeds using fungal internal transcribed spacer (ITS) gene amplicon sequencing and metagenomics. Our data showed that *Kazachstania slooffiae* (named *Candida slooffiae* before 2005 [29, 30]) was the most abundant gut fungal species in the seven breeds of weaned piglets. *K. slooffiae* inhibited lysine succinylation, and these proteins were especially enriched in the glycolysis pathway in intestinal epithelial cells. Our data indicated that *K. slooffiae*-promoted the intestinal epithelial glycolysis metabolism was mediated by lysine desuccinylation through activating sirtuin 5 (SIRT5) activity. These findings revealed that gut fungi could also play important roles in host metabolism by regulating host protein PTMs, thus providing a new perspective on the mechanism of host–gut fungi interactions. Our data also suggest a promising avenue to prevent insufficient energy supply induced by gastrointestinal disorders by manipulating the intestinal microbiota.

## Materials and methods

### Pig fecal sample collection and microbial genomic DNA extraction

All experimental procedures for pigs were approved by the Institutional Animal Care and Use Committee of the Huazhong Agricultural University (approval number: HZAUSW-2018-026). A total of 112 pigs, including 56 weaned piglets and 56 finishing pigs from seven breeds, were used in this study. Pigs of different breeds did not have the same age, diet, and environment. All weaned piglets, including eight Duroc × [Landrace × Yorkshire] (DLY), eight Tibetan miniature (TM), eight Laiwu (LW), eight Shaziling (SZL), eight Congjiang miniature (CM), eight Huanjiang miniature (HM), and eight Ningxiang (NX) piglets, ate food and drank water freely. All finishing pigs, including eight DLY, eight TM, eight LW, eight SZL, eight CM, eight HM, and eight NX pigs, ate food and drank water freely. Detailed information on the age and gender of the pigs was presented in the Additional file 11: Data S1. Detailed information on the diets was presented in the Additional file 12: Table S1-14. A total of 112 fresh feces samples from these 112 pigs were individually collected and stored in liquid nitrogen. After cleaning the anus of the pigs, sterile swabs were carefully inserted into the rectum to stimulate defecation. Fresh feces were collected immediately in sterile collection tubes. All weaned piglets were healthy and received no antibiotic, steroid, or antifungal agent from birth to the date of sample collection. All finishing pigs were healthy and received no antibiotic, steroid, or antifungal agent within 2 months prior to sample collection. Microbial genomic DNA was extracted using the procedures as described previously [31]. Briefly, fecal samples (0.2 g) were resuspended in cetyl trimethyl ammonium bromide (CTAB) buffer and lysed using beat-beading based on a FastPrep-24 bead beater (MP Bio). Three rounds of phenol:chloroform:isoamyl alcohol (V/V/V = 25: 24: 1) extraction were conducted to purify the DNA. Finally, the DNA was precipitated with a solution containing 1.5 ml ice-cold 95% ethanol and 40 μL of 3 M sodium acetate and then resuspended in Tris-ethylene diamine tetraacetic acid (EDTA) buffer. Zirconium oxide beads with 4 mm in diameter (Servicebio, G0204) were used for the bead-beating. Fecal microbial genomic DNA was used for subsequent fungal ITS gene amplicon sequencing and metagenomics.

### Fungal ITS gene amplicon sequencing and data analysis

ITS-2 region of the fungal ITS gene was amplified using the polymerase chain reaction (PCR) with the following primers: 5′–NNNNNNNNGCATCGATGAAGAACGCAGC–3′ (forward) and 5′ –TCCTCCGCTTATTGATATGC–3′ (reverse), as previously described [6, 32–38]. Agencourt AMPure XP beads were used to purify the PCR products. DNA libraries were validated as follows: the average molecule length of PCR products was determined using the Agilent 2100 bioanalyzer, and PCR products were quantified using a Qubit 2.0 Fluorometer (Thermo Fisher Scientific). Finally, qualified DNA libraries were sequenced using the Illumina Hiseq2500 system with the PE300 strategy. After filtering, clean data were obtained from raw data using SOAPnuke (v1.5.6) according to the following criteria: (1) reads containing adaptors (adapters of more than 15 bases matched to reads with maximal 3-base mismatches allowed) were discarded; (2) reads with ambiguous bases were removed; (3) reads with low complexity were removed (default: reads with 10 consecutive same bases); and (4) reads with ≥ 20% base quality (Q) ≤ 20 were removed. We used the FLASH (v1.2.11) to merge the clean reads into tags (paired-end reads merged sequences) as previously described [39–42]. Fungal tags were then clustered into amplicon sequence variants (ASVs) with 100% sequence similarity using package “DADA2” of R (v3.1.1). Fungal ASVs were taxonomically classified using the RDP classifier (v2.2), based on UNITE database (v8.2). The Chao and Shannon indexes were calculated using Mothur (v1.31.2), and rarefaction curves were drawn by the function “plot” of R. Weighted UniFrac distance in beta diversity was calculated using QIIME (v1.80), and the scatter plot of principal coordinates analysis (PCoA) was drawn using function “vegdist” of package “vegan” of R. The histograms for taxonomic composition were drawn using function “barplot2” of package “gplots” of R. The correlations between species were calculated using the algorithm “spearman” of R. A heatmap of the Spearman correlation was generated using function “heatmap.2” of package “gplots” of R. Cytoscape software was used to construct a correlation network diagram. The phylogenetic tree was constructed using FastTree (v2.1.3) and visualized using package “ape” and function “plot” of R.

### Metagenomics sequencing and data analysis

The DNA libraries for metagenomics were constructed using the Illumina TruSeq DNA PCR-Free Library Preparation Kit (Illumina, USA) and subsequently sequenced on a Hiseq Xten platform (Illumina) using the 150-bp paired-end strategy. Raw data were filtered to remove the low-quality reads, duplicate reads, and adapter contamination reads using the SOAPnuke (v1.5.6), and host genomic sequencing reads were trimmed using Bowtie2 software (v2.2.5). For each sample, the obtained high-quality clean reads were de novo-assembled using IDBA-UD software (v1.1.3) to individually generate contigs. Bowtie2 was used to align clean reads to the UNITE (v8.3) database with parameters “--sensitive -I 100 -X 800”, and thus ITS reads were extracted. The fungal ITS region is the most common genetic marker for the molecular identification of various fungal species, and it is widely accepted as the “gold standard” by fungal taxonomists [43]. The analysis of marker DNA fragments, such as 16S rDNA and 18S rDNA, derived from metagenomics has also been used to investigate bacterial and fungal communities [44, 45]. FLASH (v1.2.11) was used to merge the clean paired-end reads into tags (paired-end reads merged sequences). All the overlapping tags and non-combined reads were clustered to generate the operational taxonomic unit (OTU) sequences with 97% sequence similarity using USEARCH (v10.0.240). The OTU sequences were taxonomically classified using the “sintax” algorithm with parameter “-sintax_cutoff 0.8” based on the UNITE (v8.3) database, and these fungal OTUs were selected for downstream analysis. PCA based on the OTUs abundance was calculated, and a scatter diagram was drawn using the function “dudi.pca” of package “ade4” of R software*.* The phylogenetic tree was constructed using FastTree (v2.1.3) and visualized using package “ape” and function “plot” of R. The heatmaps for the taxonomic compositions were drawn using function “heatmap.2” of package “gplots” of R.

### Fungal strains isolation and culture

*K. slooffiae* was isolated from the feces of pigs using a yeast extract peptone dextrose (YPD) medium, containing 10 g/L yeast extract (Sangon Biotech, A515245), 20 g/L peptone (Sangon Biotech, A505247), and 20 g/L glucose (Sangon Biotech, A501991), supplemented with 1% penicillin-streptomycin (Gibco, 15140). Briefly, fresh feces (0.5 g) from DLY piglets (male, 35 days of age) were added to 5-mL sterile phosphate-buffered saline (PBS) and homogenized. Subsequently, the fecal suspension was serially diluted and spread on a YPD agar plate medium supplemented with 1% penicillin-streptomycin (Gibco, 15140). After culturing for 48 h in a constant temperature incubator under aerobic and stationary conditions at 37 °C, the single colonies grown on YPD agar plates were selected and the fungal ITS2 region of the single colony was amplified using PCR with the following primers: 5′–GCATCGATGAAGAACGCAGC–3′ (forward) and 5′ –TCCTCCGCTTATTGATATGC–3′ (reverse). *K. slooffiae* was identified based on the basic local alignment search tool (BLAST) results of the fungal ITS gene sequencing. The *K. slooffiae* isolated was cultured in the YPD growth broth medium in a constant temperature incubator under aerobic and stationary conditions at 37 °C, and the OD_600nm_ was approximately 1.0 when used for the sub-culture. *K. slooffiae* was cultured for 12 h and then used in the co-culture experiments.

### Cell culture and treatments

Intestinal porcine epithelial cell line from the jejunum (IPEC-J2) cells (a permanent intestinal cell line [46]) were cultured in cell culture dishes (Corning, 430167; bottom diameter: 83.8 mm; height: 20 mm) (not the cell culture transwell) containing DMEM/F12 medium (Gibco, 11320-033) supplemented with 1% penicillin-streptomycin (Gibco, 15140) and 10% fetal bovine serum (FBS) (Gibco, 10099) in 5% CO_2_ at 37 °C. The cells were used between passages 70 and 90. When the cells reached approximately 80–90% confluence, they were digested using trypsin-EDTA (Gibco, 25200056). Cells were counted using trypan blue staining and a light microscope. The cell density in the sub-cultured dish was 2.0 × 10^6^ cells per dish. The utilization of transwells contributes to the investigation of transepithelial transport and intestinal epithelial barrier functions [47–49]. We mainly investigated the cell metabolism and protein PTMs regulated by *K. slooffiae*. Therefore, we selected that the IPEC-J2 cells were cultured in cell culture dishes. Viable *K. slooffiae* and IPEC-J2 cells were counted using methylene blue and trypan blue staining, respectively. When the IPEC-J2 cells reached approximately 70% cell confluence (this proliferative state was suitable for studies on cell metabolism), *K. slooffiae* was added to the cell medium for 3 h or 6 h. The initial proportion of *K. slooffiae* numbers to IPEC-J2 cells numbers was 5:1 or 50:1. The number of IPEC-J2 cells that grew to 70% confluence was estimated based on the counting results of IPEC-J2 cells that were sub-cultured in parallel. The proportion of *K. slooffiae* numbers to IPEC-J2 cell numbers was chosen according to previously described co-culture procedures [50–54]. The representative pictures for the co-culture of *K. slooffiae* and IPEC-J2 cells were shown in Additional file 4: Fig. S4. After co-culture, the cells were washed with PBS to remove *K. slooffiae*. Briefly, the culture medium was removed from the cell culture dishes, and 2 mL sterile PBS was added to the cell culture dishes. Subsequently, the culture dishes were gently swirled to make the PBS cover all the cells. PBS was removed from the cell culture dishes. The cells were thus washed thrice. The cells were observed under a light microscope to ensure the removal of *K. slooffiae*. Finally, the cells were separated from the bottom of the culture dish using a cell scraper (Sangon Biotech, F619301) and resuspended in PBS solution. After centrifugation at 1000 × *g* for 10 min, the cells were collected for further western blot assay and proteomic analysis.

To investigate the roles of lysine desuccinylation in glycolysis metabolism promoted by *K. slooffiae*, sodium succinate (final concentration of 80 mM) (Sangon Biotech, A610889), which was used to increase protein lysine succinylation levels as described previously [55], and *K. slooffiae* were added into the cell medium when the IPEC-J2 cells reached approximately 70% cell confluence, and cells were then cultured for 6 h. The initial ratio of *K. slooffiae* numbers to IPEC-J2 cell numbers was 50:1. The number of IPEC-J2 cells was estimated based on the counting results of cells that were sub-cultured in parallel as described above. To investigate the role of SIRT5 in glycolysis metabolism promoted by *K. slooffiae*, IPEC-J2 cells were cultured for 6 h with *K. slooffiae* and either nicotinamide (NAM, final concentration of 5 mM) (Sigma, N0636), that inhibits the activity of sirtuins (SIRTs) as previously described [56, 57], or NRD167 (final concentration of 10 μM) (Selleck, S9903), that specifically inhibits SIRT5 activity as previously described [58], added to cell culture medium. Concentrations of these drugs were chosen according to previously described procedures [55–58]. After co-culture with *K. slooffiae* for 6 h, the cells were immediately washed to remove *K. slooffiae* and collected for western blot assay. Glucose consumption, lactate production, and adenosine triphosphate (ATP) content were measured, as described below.

To investigate the roles of *K. slooffiae*-derived metabolites in glycolysis mediated by lysine desuccinylation, 5′-methylthioadenosine (final concentration of 50 μM or 100 μM) (Aladdin, D168979) was added to the cell medium, and IPEC-J2 cells were cultured for 6 h. In addition, *K. slooffiae* was added to the cell medium and IPEC-J2 cells were cultured for 6 h as a positive control. Finally, the cells were collected for western blot analysis. Glucose consumption, lactate production, ATP content, cAMP levels, and SIRTs activity were measured as described below. The concentrations of 5′-methylthioadenosine were chosen according to previously described procedures [59, 60].

### Western blotting

The cells were lysed using RIPA lysis buffer (Sangon Biotech, C500005) supplemented with 3 μM trichostatin A (TSA) (MCE, HY-15144) and 50 mM NAM to prepare whole-cell lysates (WCLs). Considering that HDACs class I/II and SIRTs are widely involved in PTMs, the TSA (an HDAC class I/II inhibitor [61]) and NAM (an SIRT inhibitor [62]) were used to prevent the alteration of PTMs after the cells were lysed, as previously described [63, 64]. Western blot assay was conducted as previously described [65]. Briefly, protein samples were separated by sodium dodecyl sulfate-polyacrylamide gel electrophoresis (SDS-PAGE) and transferred to polyvinylidene fluoride (PDVF) membranes. PVDF membranes were incubated with fat-free milk, primary antibody, and horseradish peroxidase (HRP)-conjugated secondary antibody in sequence. Finally, signals were measured by an enzyme-linked enhanced chemiluminescence (ECL) reagent (Thermo Scientific, 34580). Primary antibodies, including anti-succinyllysine (PTM BIO, PTM-419, dilution rate: 1: 500), anti-ubiquitin (PTM BIO, PTM-1107, dilution rate: 1: 2,000), anti-acetyllysine (PTM BIO, PTM-101, dilution rate: 1: 1000), anti-crotonyllysine (PTM BIO, PTM-502, dilution rate: 1: 1000), anti-2-hydroxyisobutyryllysine (PTM BIO, PTM-802, dilution rate: 1: 750), and anti-β-tubulin (Proteintech, 66240-1-Ig, dilution rate: 1: 20,000), were used in this assay. The HRP-conjugated secondary antibody (goat anti-mouse) (Cell Signaling Technology, 7076S, dilution rate: 1: 5000) was used in this assay. PVDF membranes were incubated with primary antibody at 4 °C for 12 h and HRP-conjugated secondary antibody at room temperature for 1.5 h.

### Proteomics analysis for protein lysine succinylation

The cells were collected and then resuspended in a buffer containing 8 M urea (Sigma-Aldrich, U5378), 1% protease inhibitor cocktail (MedChemExpress, HY-K0010), 3 μM TSA (MedChemExpress, HY-15144), and 50 mM NAM (Sigma-Aldrich, N0636). These cells were lysed by ultrasonication thrice on ice using a high-intensity ultrasonic processor (Scientz), and the supernatant was obtained after centrifugation at 12,000 × *g* for 10 min at 4 °C. Protein supernatant was processed with 5 mM dithiothreitol (Sigma-Aldrich, D9163) and 11 mM iodoacetamide (Sigma-Aldrich, V900335). Subsequently, the protein supernatant was diluted with 0.2 M triethyl ammonium bicarbonate (TEAB) buffer (Sigma-Aldrich, T7408) and the volume ratio of protein supernatant to TEAB buffer was 1:4. The diluted protein supernatant was digested twice with 20 ng/ μL trypsin (Promega, V5117) at 37 °C for 12 h. The tryptic peptides were dissolved in the immunoprecipitation (IP) buffer (Thermo Fisher Scientific, 87787) and then incubated with anti-succinyllysine antibody-conjugated agarose beads (PTM BIO, PTM-402) on a shaker (Servicebio, DS-3D100) with gentle shaking at 4 °C for 12 h. After washing four times with IP buffer, twice with ddH_2_O, and once with 0.1% trifluoroacetic acid (Sigma-Aldrich, 302031), the bound peptides were eluted. The peptides were desalted using C18 ZipTips and separated using an EASY-nLC 1000 UPLC system. Finally, separated peptides were subjected to nanospray ionization (NSI) source followed by tandem mass spectrometry (MS/MS) in Q ExactiveTM Plus (Thermo) coupled online to the UPLC system.

The MS/MS data were processed using the MaxQuant search engine and searched against the Uniprot database (Sus_scrofa_9823_PR_20180816). Trypsin/P was chosen as the cleavage method, and two missing cleavage sites were allowed. In the first search to correct the mass axis, 20 ppm was chosen as the mass tolerance for the precursor ions. In the main search used to obtain the candidate peptides for subsequent matching, 5 ppm was chosen as the mass tolerance for the precursor ions. Mass tolerance was set to 0.02 Da for the fragment ions. A false discovery rate (FDR) of < 1% was set. The mean intensity for each modification site in the three replicates was calculated, and the fold change was calculated as the mean intensity ratio for each modification site in the two groups. Student’s *t*-test was conducted to calculate *p*-values. A ratio of 1.5 fold-change (>1.50 or <0.67) with a *p*-value < 0.05 was chosen as the cutoff for up- or down-regulation in lysine succinylation. We used the PCA by the function “prcomp” with parameter “scale = true” of package “prcomp” of R (v3.1.1) software, relative standard deviation (RSD) analysis by package “ggplot” of R, and Pearson’s correlation coefficient by package “corrgram” of the R based on function “corrgram” to evaluate the repeatability of quantitative analysis. Volcano plot analysis by package “ggplot” of the R was used to show the differential sites with lysine succinylation. MoMo software with parameter “*p*-value<0.000001 and minimum number of occurrences = 20” was used for motif analysis. Gene ontology (GO) annotations with parameter “-goterms -iprlookup -pa” were conducted using InterProScan. KEGG annotation was performed using the KAAS and KEGG Mapper. Enrichment analyses of the GO and KEGG were conducted using the Perl module, and *p*-value was calculated using Fisher’s exact test.

### Label-free quantitative proteomics and data analysis

The WCLs were prepared, and the protein solution was digested using the procedures described above. Subsequently, tryptic peptides were fractionated by high pH reverse-phase HPLC using a Thermo BetaSil C18 column (Thermo Fisher Scientific, 70105-254630). Tryptic peptides were then separated using an EASY-nLC 1000 UPLC system. Separated peptides were then subjected to an NSI source, followed by MS/MS in Q ExactiveTM Plus coupled online to the UPLC system. MS/MS data were processed and searched using the procedures described above. The mean intensity for each protein in the three replicates was calculated, and the fold change was calculated as the mean intensity ratio for each protein in the two groups. Student’s *t*-test was used to calculate *p*-values. A ratio of 1.5 fold-change (>1.50 or <0.67) with a *p*-value < 0.05 was chosen as the cutoff for protein up- or down-regulation. Volcano plot analysis by package “ggplot” of R software was used to show the differentially expressed proteins.

### Measurements of glucose consumption, lactate production, ATP content, cAMP levels, and SIRTs activity in cells

The glucose and lactate levels in the cell medium were examined using glucose (Sangon Biotech, D799408) and lactic acid (Sangon Biotech, D799851) content assay kits, respectively. The protein concentration of WCLs was measured using a kit (Beyotime, P0012). Glucose consumption and lactate production were normalized to the final protein content of WCLs. ATP levels were measured using an assay kit (Thermo Scientific, A22066), and ATP content was normalized to the protein concentration of WCLs. cAMP levels were examined using an ELISA kit (MM-0544, MEIMIAN), and cAMP levels were normalized to the protein concentration of WCLs. The total activity of SIRTs was examined using a SIRT activity assay kit (Epigentek, P-4036). Briefly, IPEC-J2 cells were lysed by NP-40 lysis buffer (Beyotime, P0013F), and the protein solution was added to the wells of the SIRT substrate plate. One microliter of TSA (HDAC class I/II inhibitor, 50 μM) and 1 μL nicotinamide adenine dinucleotide (NAD^+^) (SIRT cofactor, 7.5 mM) was added to each well. TSA was used to inhibit the HDAC class I/II activity to rule out the effects of HDAC class I/II [61]. NAD^+^ cofactor is required for SIRTs activity [66]. TSA and NAD^+^ were included in the SIRT activity assay kit (Epigentek, P-4036). After incubation for 90 min at 37 °C, the reaction solution was removed from each well. Subsequently, the capture antibody solution was added to the wells and incubated at room temperature for 60 min, and the detection antibody solution was added to the wells and incubated for 30 min. The detection solution was added to the wells to develop color, and the reaction was stopped using a stop solution. Finally, the OD450 nm was measured. The activity levels of SIRTs were normalized to the protein concentration of the WCLs.

### Identification of fungal metabolites using untargeted metabolomics

*K. slooffiae* were cultured in the cell culture dishes containing DMEM/F12 medium supplemented with 1% penicillin-streptomycin and 10% FBS in 5% CO_2_ at 37 °C. Since the co-culture of IPEC-J2 cells and *K. slooffiae* was conducted in DMEM/F12 medium, we chose DMEM/F12 medium as the culture medium to identify the *K. slooffiae*-derived metabolites that may mediate the cell metabolism in IPEC-J2 cells. After culturing for 6 h, the culture supernatant of *K. slooffiae* was collected by centrifugation at 6080 × *g*. Global metabolites of the culture supernatant of *K. slooffiae* and the culture medium that was not inoculated with strain were analyzed using UPLC combined with MS and the detailed procedures were previously described [67]. Briefly, samples were extracted and analyzed using UPLC and quadrupole-time of flight (QTOF)-MS/MS to identify metabolites based on public databases, including Metline, KEGG, HMDB, Massbank, and Nist-MSMS. The differential metabolites between the two groups were identified using the criteria of variable importance in projection (VIP) ≥1 from the orthogonal partial least squares discrimination analysis (OPLS-DA), absolute Log_2_ (fold-change) ≥1, and *p*-value < 0.05. PCA was performed using R (v3.1.1) software to show the difference among samples, and the function “prcomp” with parameter “scale = true” of package “prcomp” of the R was used. The volcano plot was drawn using package “ggplot” of the R to show the differential metabolites between the two groups.

### Oral administration of fungal strains in germ-free (GF) mice

All experimental procedures involving mice were approved by the Institutional Animal Care and Use Committee of Huazhong Agricultural University (approval number: HZAUMO-2019-088). Eighteen GF Kunming mice (male, 3 weeks of age) of similar weights (12 ± 0.12 g) were used. GF Kunming mice were bred in the Department of Laboratory Animal Science at the Army Medical University in Chongqing, China. The mice were housed in sterile isolators (temperature, 25 ± 2°C; relative humidity, 45–60%; lighting cycle, 12 h/day, Beijing time 06:30–18:30). GF mice in group 1 (Ctrl, *n* = 10) were treated with a vehicle (sterile PBS, 150 μL) through oral gavage every 3 days from 3 to 8 weeks of age. GF mice in group 2 (KS, *n* = 8) were treated with a suspension (150 μL, 10^8^ CFU/mL) containing *K. slooffiae* by oral gavage every 3 days from 3 to 8 weeks of age. To ensure the similar mean weights of mice between the two groups so far as possible, a total of ten mice and eight mice were randomly divided into two groups. The difference in the number of mice between the two groups did not adversely affect the statistical analysis, such as Student’s *t*-test. All mice had free access to the same sterile food (Beijing HFK Bioscience, 1025) treated with Cobalt-60 gamma irradiation sterilization and sterile autoclaved water. The GF mice in each group were housed in individual sterile isolators. GF mouse experiments were conducted at 3–8 weeks of age. After sacrifice, the jejunal contents and feces from the GF mice were collected. *K. slooffiae* was isolated from the jejunal contents and feces of GF mice that were orally administrated *K. slooffiae* using the detailed isolation procedures for pig feces and identified as described above. In addition, three GF Kunming mice (male, 8 weeks of age) of similar weights (44.47 ± 0.45 g) and three specific pathogen-free (SPF) Kunming mice (male, 8 weeks of age) of similar weights (44.6 ± 0.59 g) were used to evaluate the effect of GF status on protein succinylation in intestinal epithelial cells, and these six mice had free access to same food and water as described above.

After sacrifice, jejunal epithelial cells were isolated from GF mice using previously described methods [65]. Briefly, the middle part of the jejunum, approximately 2–3 cm in length, was taken out and everted. The jejunum tissue was divided into 3 × 3 mm pieces, approximately, and gently washed in Hank’s balanced salt solution (HBSS) (Gibco, 14025) at 4 °C for 5 min using a shaker (Servicebio, DS-3D100). Subsequently, the jejunal tissues were incubated in an ice-cold chelating buffer (27 mM trisodium citrate (A501293), 5 mM Na_2_HPO4 (A501727), 96 mM NaCl (A501218), 8 mM KH_2_PO4 (A501211), 1.5 mM KCl (A501159), 0.5 mM dithiothreitol (A620058), 55 mM D-sorbitol (A610491), and 44 mM sucrose (A502792)) with constant shaking at 100 rmp for 45 min in a shaker (Servicebio, DS-3D100). All reagents for the chelating buffer were purchased from the Sangon Biotech (Shanghai, China). We removed the remaining intestinal tissue. After centrifugation at 1000 × *g* for 10 min at 4 °C, the cells isolated were obtained. Finally, the cells were washed three times with PBS. Isolated jejunal epithelial cells were used for western blot and cell culture. Collagen I-coated cell culture dishes contribute to the adhesion of primary cells. The isolated jejunal epithelial cells were cultured in the collagen I-coated cell culture dishes (Corning, 354450; bottom diameter: 83.8 mm; height: 20 mm) containing DMEM/F12 medium (Gibco, 11320-033) in 5% CO_2_ at 37 °C. DMEM/F12 medium was supplemented with 1% penicillin-streptomycin-amphotericin B (Gibco, 15240), and 10% FBS (Gibco, 10099). Additionally, 20 ng/mL epidermal growth factor (EGF) (Sigma-Aldrich, E4127) and 50 μM sodium orthovanadate (Sigma-Aldrich, 567540) were added to the medium. EGF contributes to the growth of isolated jejunal epithelial cells. Sodium orthovanadate supplementation contributes to decreasing the cellular sensitivity to anoikis, thereby enhancing cell survival in primary cell culture, as described previously [68, 69]. Glucose consumption, lactate production, and ATP content were measured, as described above.

### Oral administration of fungal strains in pigs and comparative analysis of intestinal protein lysine succinylation between the two pig breeds

All experimental procedures involving pigs were approved by the Institutional Animal Care and Use Committee of the Huazhong Agricultural University (approval number: HZAUSW-2022-0014). Commercial DLY pig is the most widely bred pig breed [70, 71]. Neonatal animals are appropriate for studies on gut microbial function because their gut microbiota exhibits higher plasticity and adaptability than that in adults [72]. Thus, a total of 20 neonatal DLY male piglets with similar weights (1.5 ± 0.09 kg) were used. The piglets in group 1 (Ctrl, *n* = 10) were treated daily with sterile PBS (2 mL) via oral gavage. The piglets in group 2 (KS, *n* = 10) were treated daily with a suspension of *K. slooffiae* (2 mL, 10^8^ CFU/mL) by oral gavage. The dosage of *K. slooffiae* in pigs was chosen according to previously described procedures for probiotic yeasts [73–79]. The experiments were conducted from birth to 3 weeks of age, and the piglets were suckling the breast. After sacrifice, jejunal epithelial cells were isolated from piglets using previously described methods [65], and detailed procedures were described above. Isolated jejunal epithelial cells were used for western blot assays and further cell culture. The detailed procedures for cell culture were described above.

Five CM finishing pigs and five DLY finishing pigs were used to compare the intestinal protein lysine succinylation levels and glycolysis. Detailed information on the age and gender of the pigs is presented in Additional file 11: Data S1. Detailed information on the diets is presented in Additional file 12: Table S8 and Table S12. All finishing pigs were healthy and received no antibiotic, steroid, or antifungal agent within 2 months before sample collection. After sacrifice, jejunal epithelial cells were isolated from the pigs using previously described methods [65], and detailed procedures were described above. Isolated jejunal epithelial cells were used for western blot assays and further cell culture. The detailed procedures for cell culture were described above.

### Analysis of intestinal histological morphology

Hematoxylin and eosin (H&E) staining was conducted to show intestinal histological morphology following previously described procedures [80]. Briefly, duodenum, jejunum, and ileum segments were fixed in a 4% paraformaldehyde solution. Subsequently, the intestinal segments were embedded in paraffin and cut into 5-μm thick sections. The sections were sequentially stained with hematoxylin and eosin. Intestinal images were obtained using a light microscope. Villus height and crypt depth were measured according to at least 50 representative villi and crypts per pig, as mentioned previously [81].

### Statistical analysis

**Fig. 1:**
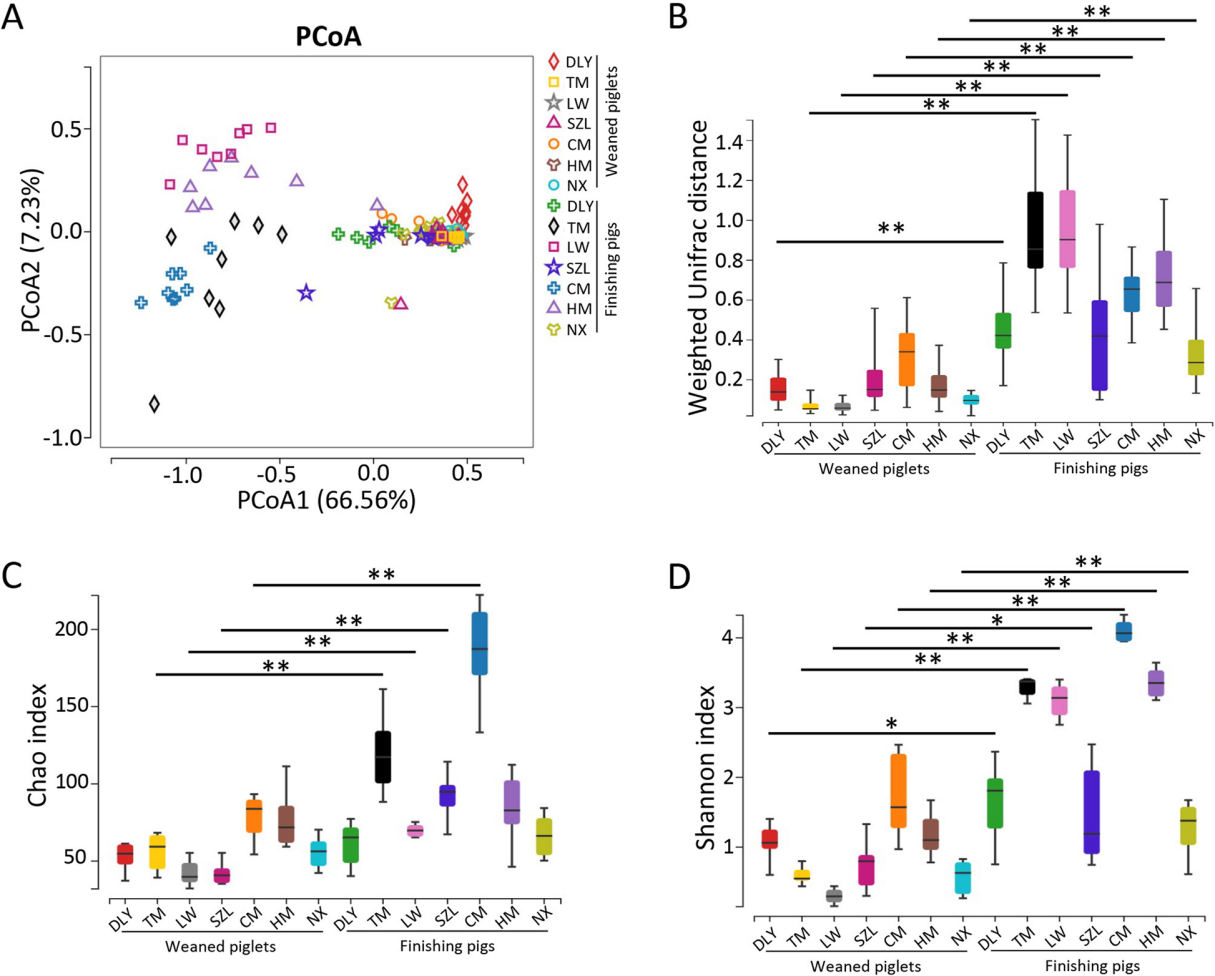
Diversities of gut fungal communities in pigs revealed by ITS gene amplicon sequencing. **A** Scatterplot from PCoA of the fungal communities based on weighted UniFrac distance. **B** Weighted UniFrac distance of fungal communities. **C** Analysis of fungal Chao index. **D** Analysis of fungal Shannon index. Data are presented as mean ± SEM (*n* = 8). Kruskal-Wallis test and adjustments for multiple comparisons were conducted. **p < 0.01, *p < 0.05

Both GraphPad Prism (v6.0c) and R (v3.1.1) software were used for statistical analyses. The Kruskal-Wallis test was conducted in Fig. 1B–D. One-way analysis of variance (ANOVA) was conducted in Figs. 4A–C, 6B–E, J–M, and 7F–K. Two-way ANOVA was conducted in Figs. 4G and 8N–P. Student’s *t*-test was conducted in Figs. 5D–F, 6H, and 8B–F, H–L, Fig. S8B, and S10B-E. A ratio of 1.5-fold (>1.50 or <0.67) with a *p*-value < 0.05 was chosen as the cutoff for up- or down-regulation in lysine succinylation in Fig. 4J and K. A ratio of 1.5-fold (>1.50 or <0.67) with a *p*-value < 0.05 was chosen as the cutoff for protein up- or downregulation in Fig. 6F. The differential metabolites between the two groups were identified using the criteria of VIP ≥1 from the OPLS-DA, absolute Log_2_ (fold-change) ≥1, and *p*-value < 0.05 in Fig. 7C and D. Detailed statistical methods are also provided in the figure legends. Statistical significance was set at *p*-value < 0.05.

## Results

### A landscape of pig gut fungal communities

We analyzed pig gut fungal communities to mine the core-predominant gut fungal species. We collected fecal samples from 112 pigs belonging to seven representative pig breeds in China (Additional file 1: Fig. S1A). These pigs included commercial DLY weaned piglets, native TM weaned piglets, native LW weaned piglets, native SZL weaned piglets, native CM weaned piglets, native HM weaned piglets, native NX weaned piglets, commercial DLY finishing pigs, native TM finishing pigs, native LW finishing pigs, native SZL finishing pigs, native CM finishing pigs, native HM finishing pigs, and native NX finishing pigs. Pigs of different breeds did not have the same age, diet, and environment. The results of the fungal ITS gene amplicon sequencing survey showed that sequencing depth was sufficient to detect almost all fungal species, as evidenced by the rarefaction curves (Additional file 1: Fig. S1B). The PCoA based on weighted UniFrac distance also indicated that gut fungal beta diversities in weaned piglets were similar among the seven pig breeds, whereas gut fungal beta diversities in finishing pigs were distinct among the seven pig breeds (Fig. 1A). The gut fungal beta diversities in DLY, SZL, and NX finishing pigs were similar to those in weaned piglets (Fig. 1A). The results of weighted UniFrac distances on beta diversity further supported the results of the PCoA and suggested differences in beta diversity between finishing pigs and their corresponding weaned piglets (Fig. 1B). Alpha diversity analysis showed that Chao indexes in TM, LW, SZL, and CM finishing pigs were higher than those in the corresponding TM, LW, SZL, and CM weaned piglets, respectively (Fig. 1C). The Shannon indexes in finishing pigs of all the seven pig breeds were higher than those of the corresponding weaned piglets (Fig. 1D). Thus, gut fungal alpha diversities in finishing pigs were higher than those in corresponding weaned piglets, suggesting that gut fungal diversity increased with the age of pigs.

### *K. slooffiae* is the most abundant gut fungal species in pigs

**Fig. 2:**
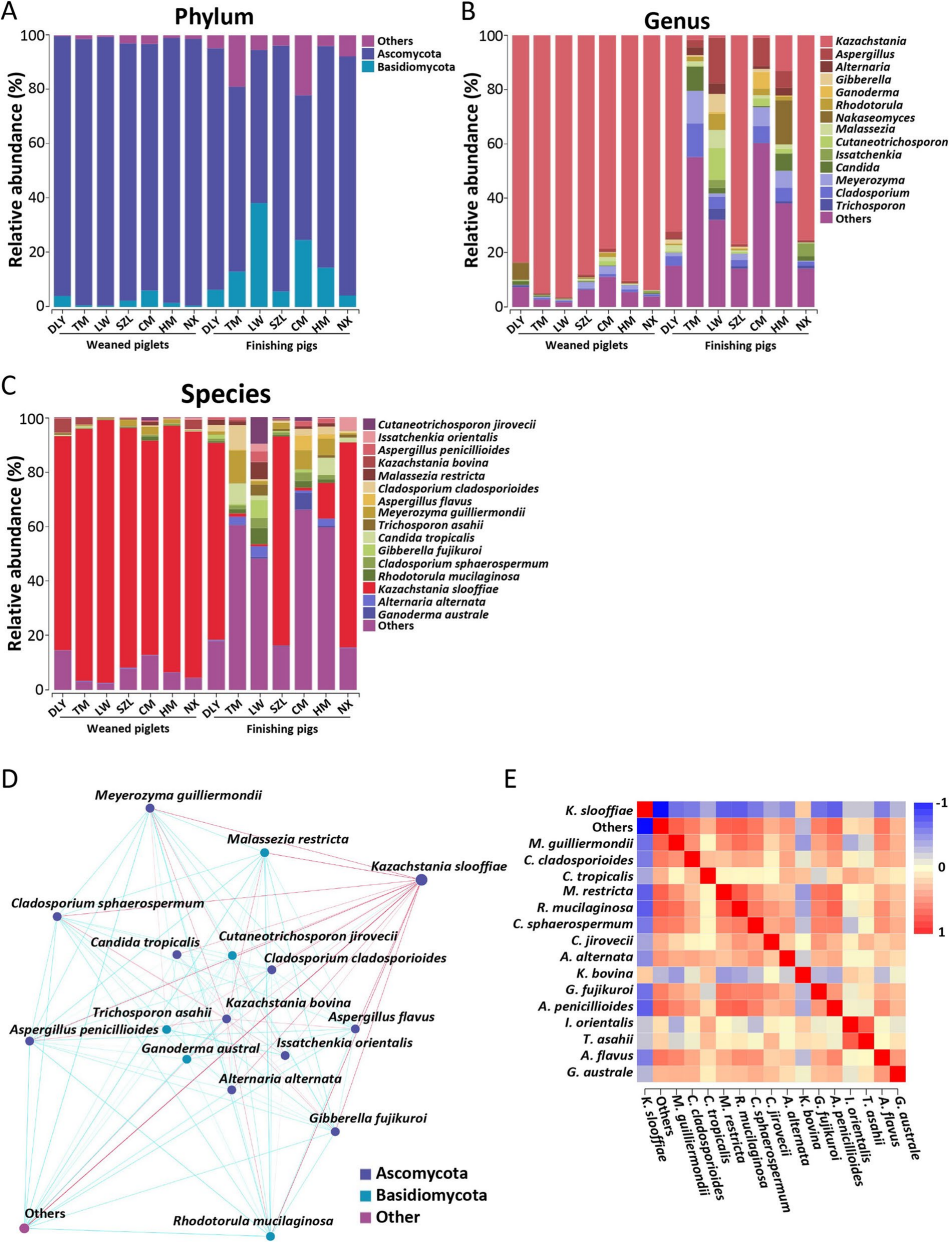
Taxonomic compositions of gut fungal communities revealed by ITS gene amplicon sequencing. **A** Gut fungal taxonomic compositions at the phylum level (DLY, Duroc × [Landrace × Yorkshire]; TM, Tibetan miniature; LW, Laiwu; SZL, Shaziling; CM, Congjiang miniature; HM, Huanjiang miniature; and NX, Ningxiang). The results are shown according to the groups. **B** Gut fungal taxonomic compositions at genus level. The results are shown according to the groups. **C** Gut fungal taxonomic compositions at species level. The results are shown according to the groups. **D** Network analysis of gut fungal species using Cytoscape. **E** Heatmap analysis of gut fungal species using Spearman correlation

Next, we investigated the taxonomic composition of gut fungi in pigs. The results showed that gut fungi mainly belonged to two phyla, Ascomycota, and Basidiomycota, with Ascomycota being the most abundant (Fig. 2A). The *Kazachstania* was the most abundant fungal genus in weaned piglets and DLY, SZL, and NX finishing pigs (Fig. 2B). *Cladosporium* was the most abundant fungal genus in the TM finishing pigs (Fig. 2B). *Nakaseomyces* was the most abundant fungal genus in the HM finishing pigs (Fig. 2B). *Aspergillus* was the most abundant fungal genus in the LW and CM finishing pigs (Fig. 2B). At the species level, the results showed that *K. slooffiae* was the most abundant gut fungal species in weaned piglets, DLY finishing pigs, SZL finishing pigs, and NX finishing pigs (Fig. 2C). The mean relative abundance of *K. slooffiae* was above 78% in the DLY, TM, LW, SZL, CM, HM, and NX weaned piglets (Fig. 2C). The mean relative abundance of *K. slooffiae* in DLY, TM, LW, SZL, CM, HM, and NX finishing pigs was 72.3%, 1.23%, 0.82%, 76.6%, 1.01%, 13.1%, and 75.2%, respectively (Fig. 2C). *Meyerozyma guilliermondii* was the most abundant fungal species in the TM and CM finishing pigs (Fig. 2C). The mean relative abundance of *M. guilliermondii* was 12.0% and 6.9% in TM and CM finishing pigs, respectively. *Cutaneotrichosporon jirovecii* was the most abundant fungal species in the LW finishing pigs (Fig. 2C). The mean relative abundance of *C. jirovecii* was 9.7% in LW finishing pigs. The species-level taxonomic histograms based on the 112 samples further supported these results that *K. slooffiae* dominated the pig gut fungal communities (Additional file 2: Fig. S2A). The network diagram and heatmap based on the correlation analysis indicated that *K. slooffiae* is the keystone fungal species with the highest connectivity degree in the gut fungal communities of pigs (Fig. 2D and E). Phylogenetic tree analysis suggested a close evolutionary relationship between the genus *Kazachstania* and the genera *Trichobolus*, *Spiroplana*, and *Simplicillium* (Additional file 2: Fig. S2B).

**Fig. 3:**
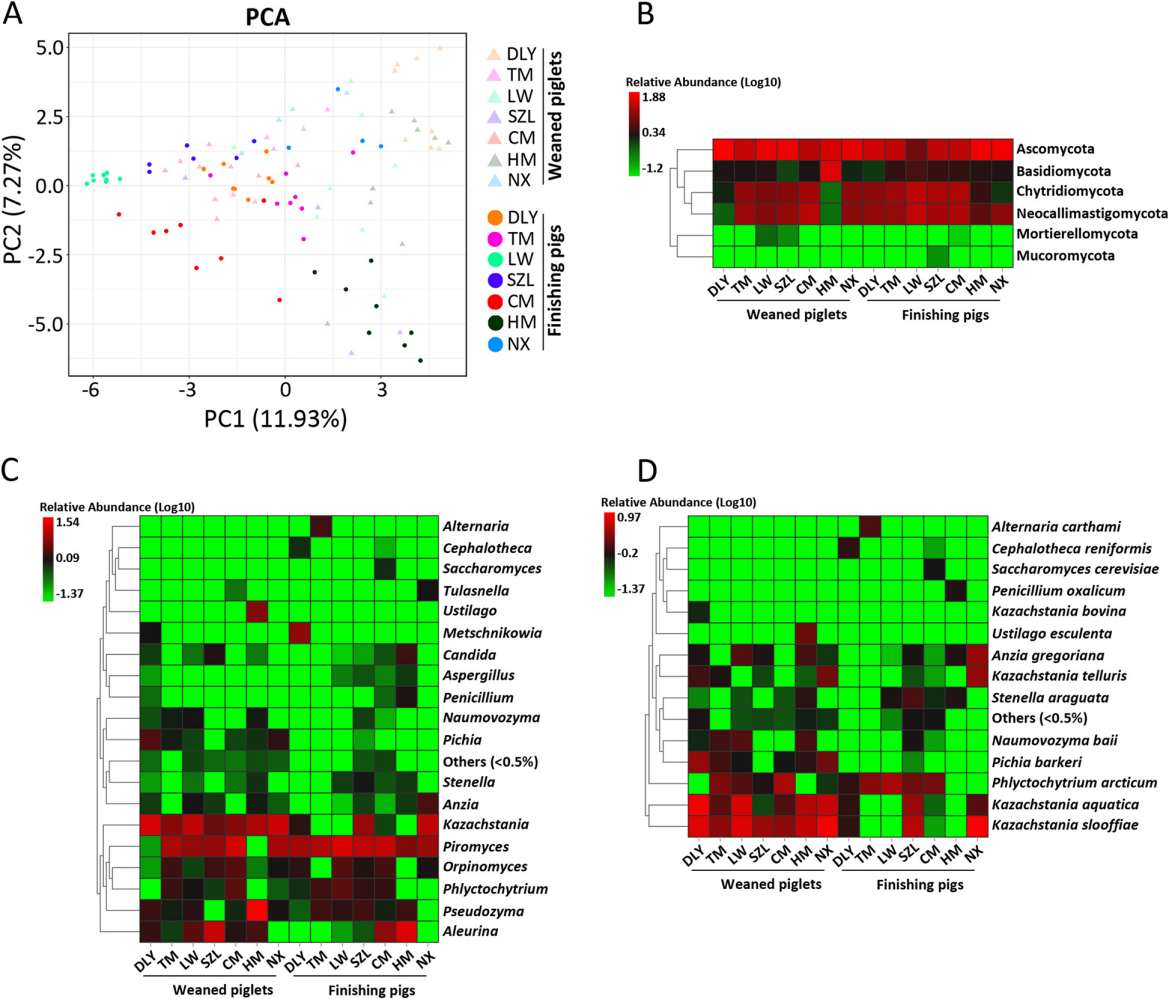
Gut fungal communities revealed by metagenomics. **A** Scatter diagram from PCA of gut fungal communities. **B** Heatmap analysis of gut fungal taxonomic compositions at phylum level. **C** Heatmap analysis of gut fungal taxonomic compositions at genus level. **D** Heatmap analysis of gut fungal taxonomic compositions at species level

To further confirm that *K. slooffiae* dominated the pig gut fungal communities, we used the metagenomics to analyze the gut fungal composition. Using de novo assembly, we identified 11,418,273 (11.4 million) non-redundant (NR) pig gut microbial genes. The gene length of NR genes mainly ranged from 200 to 1500 bp (Additional file 3: Fig. S3A). Scatter diagram of PCA showed that the fungal compositions were distinct among the seven pig breeds (Fig. 3A). The results of the taxonomic compositions of the gut fungi showed that Ascomycota was the most abundant phylum (Fig. 3B). Gut fungi mainly belonged to two genera: *Kazachstania* and *Piromyces* (Fig. 3C). *K. slooffiae* was the most abundant gut fungal species in TM, LW, SZL, HM, and NX weaned piglets and in DLY, SZL, and NX finishing pigs, as shown in the heatmap (Fig. 3D). Phylogenetic tree analysis suggested a close evolutionary relationship between the genus *Kazachstania* and the genera *Xenasmatella*, *Pichia*, *Ustilago*, and *Penicillium* (Additional file 3: Fig. S3B). Thus, our data showed that *K. slooffiae* is the most abundant gut fungal species in weaned piglets, based on fungal ITS gene amplicon sequencing and metagenomics.

### *K. slooffiae* decreases lysine succinylation in intestinal epithelial cells

**Fig. 4:**
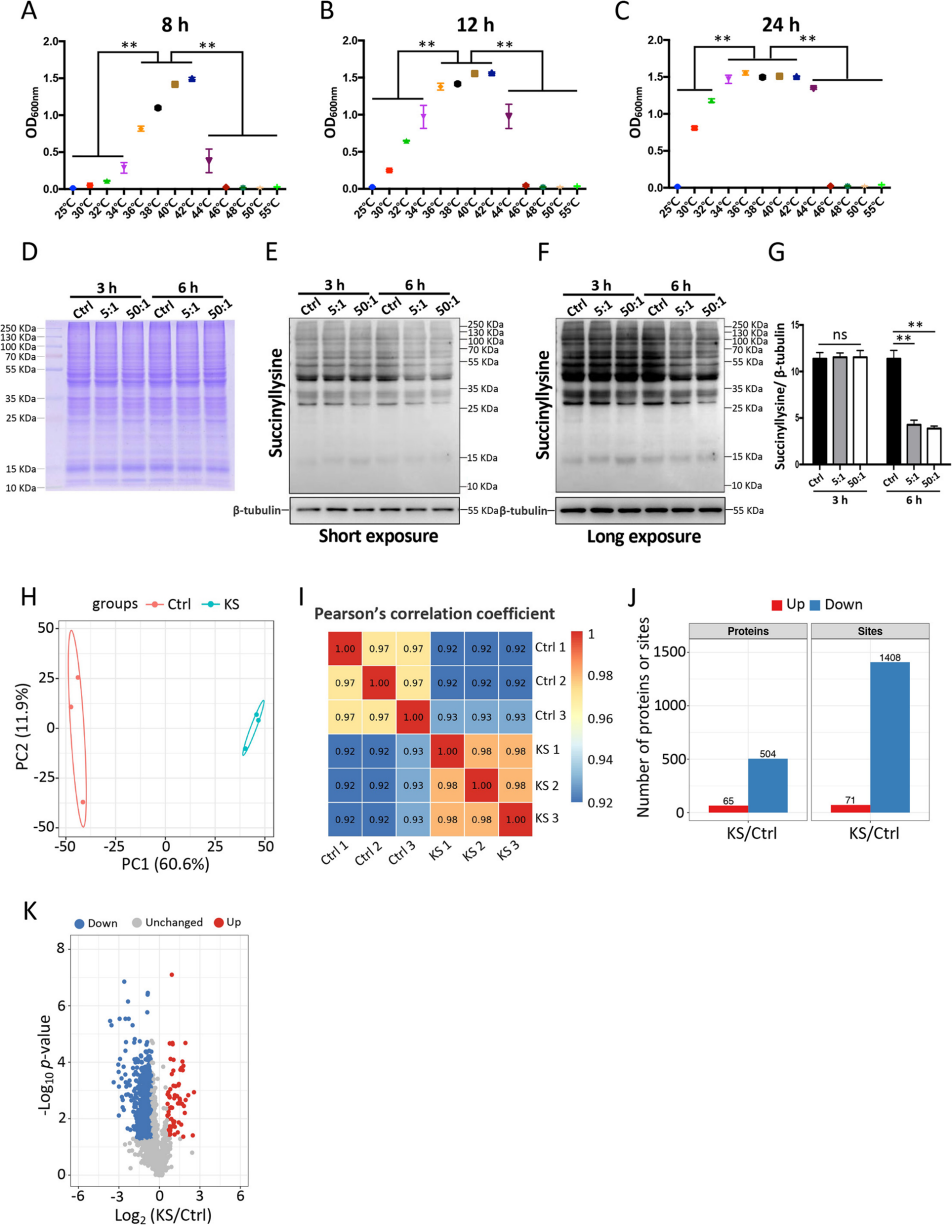
*K. slooffiae* decreases lysine succinylation levels in IPEC-J2 cells. **A**–**C** OD_600nm_ of fungal culture suspensions after culturing for 8 h (**A**), 12 h (**B**), and 24 h (**C**), respectively. **D** Coomassie blue staining of protein samples from whole-cell lysates of IPEC-J2 cells. **E**–**G** Representative western blots of lysine-succinylated proteins and β-tubulin in IPEC-J2 cells using a pan anti-succinyllysine monoclonal antibody and an anti-β-tubulin antibody, respectively (**E** and **F**). **G** Quantification of lysine-succinylated proteins levels normalized to β-tubulin levels from the blots shown in **F**. **H**, **I** Analysis of the lysine succinylome based on PCA (**H**) and Pearson’s correlation coefficient (**I**). **J** Statistical analysis of differential proteins and sites with lysine succinylation comparing the *K. slooffiae* (KS) group with the control (Ctrl) group. Up indicates that lysine succinylation levels of the proteins or sites in KS group were higher than those in Ctrl group. Down indicates that lysine succinylation levels of the proteins or sites in KS group were lower than those in Ctrl group. **K** Volcano plots showing differential sites with lysine succinylation comparing the KS group with the Ctrl group. Data are shown as mean ± SEM and evaluated by one-way analysis of variance (ANOVA) with adjustment for multiple comparisons in **A**–**C** (*n* = 4). Data are presented as the mean ± SEM and were evaluated by two-way ANOVA with adjustment for multiple comparisons in **G** (*n* = 3). **p < 0.01. A ratio of 1.5-fold (>1.50 or <0.67) with a *p*-value < 0.05 was chosen as the cutoff for up- or downregulation in lysine succinylation in **J** and **K** (*n* = 3)

To investigate the contribution of *K. slooffiae* to host, we isolated the *K. slooffiae* from the fresh feces of DLY piglets (male, 35 days of age). Interestingly, the suitable growth temperature for *K. slooffiae* was 36 to 42 °C (Fig. 4A–C), suggesting that *K. slooffiae* can adapt to the temperature in the gut lumen of pigs. Given the important roles of interactions of gut microbes with intestinal epithelium in the host health [1], we next evaluated the potential regulatory function of *K. slooffiae* on intestinal epithelial cells of pigs. Jejunum is the middle part of the small intestine that makes up approximately 93% of the surface area of the whole intestinal tract [82, 83]. The jejunum has a histological pattern similar to the entire small intestine, and it has a critical role in the immune responses, digestion, and absorption of main nutrients, such as water, carbohydrates, fatty acids, proteins, vitamins, and minerals [82–84]. The jejunal epithelial cells have been widely used to investigate that host-microbe interactions [32, 46]. Previous studies have showed that *K. slooffiae* (named as *Candida slooffiae* before 2005 [29, 30]) was isolated from the contents of small intestine (duodenum, jejunum, and ileum), caecum, and rectum of pigs, suggesting that *K. slooffiae* colonizes the jejunum of pigs [85, 86]. Live *K. slooffiae* was added to the culture medium of IPEC-J2 cells, and the co-culture was performed for 3 h or 6 h (Additional file 4: Fig. S4). Considering that PTMs of proteins play critical roles in cell metabolism, innate immunity, and pathogenesis [18], we used western blot assay with pan anti-succinyllysine, pan anti-crotonyllysine, pan anti-2-hydroxyisobutyryllysine, pan anti-acetyllysine, and pan anti-ubiquitination antibodies to preliminarily investigate the potential types of PTMs in IPEC-J2 cells that could be affected by *K. slooffiae* (Fig. 4D–G and Additional file 5: Fig. S5A-D). The results indicated that lysine succinylation levels in IPEC-J2 cells were decreased by *K. slooffiae* when co-culture was performed for 6 h. The decrease was observed when the initial proportion of *K. slooffiae* numbers to IPEC-J2 cells numbers was either 5:1 or 50:1 (Fig. 4D–G and Additional file 5: Fig. S5A-D).

### Lysine succinylome in intestinal epithelial cells is altered by *K. slooffiae*

We used a quantitative proteomics approach to compare intestinal epithelial cellular lysine succinylation between control (Ctrl) and *K. slooffiae* (KS) groups in the co-culture experiment. The initial proportion of *K. slooffiae* to IPEC-J2 cells numbers was 50:1 at the beginning of co-culture, and the co-culture was performed for 6 h. The PCA results suggested an obvious difference in lysine succinylation between the Ctrl and KS groups (Fig. 4H). The RSD analysis suggested strong repeatability of the quantitative analysis (Additional file 5: Fig. S5E), and Pearson’s correlation coefficient analysis suggested a positive correlation within groups (Fig. 4I). 5371 lysine succinylation sites in 1273 proteins were quantified totally. The succinylation levels of 71 lysine sites on 65 proteins were significantly increased, whereas the succinylation levels of 1408 lysine sites on 504 proteins were decreased after *K. slooffiae* treatment (Fig. 4J and K). Motif analysis further showed that several motifs were enriched with lysine succinylation sites (Additional file 6: Fig. S6).

### *K. slooffiae* promotes intestinal epithelial glycolysis metabolism mediated by lysine desuccinylation

**Fig. 5:**
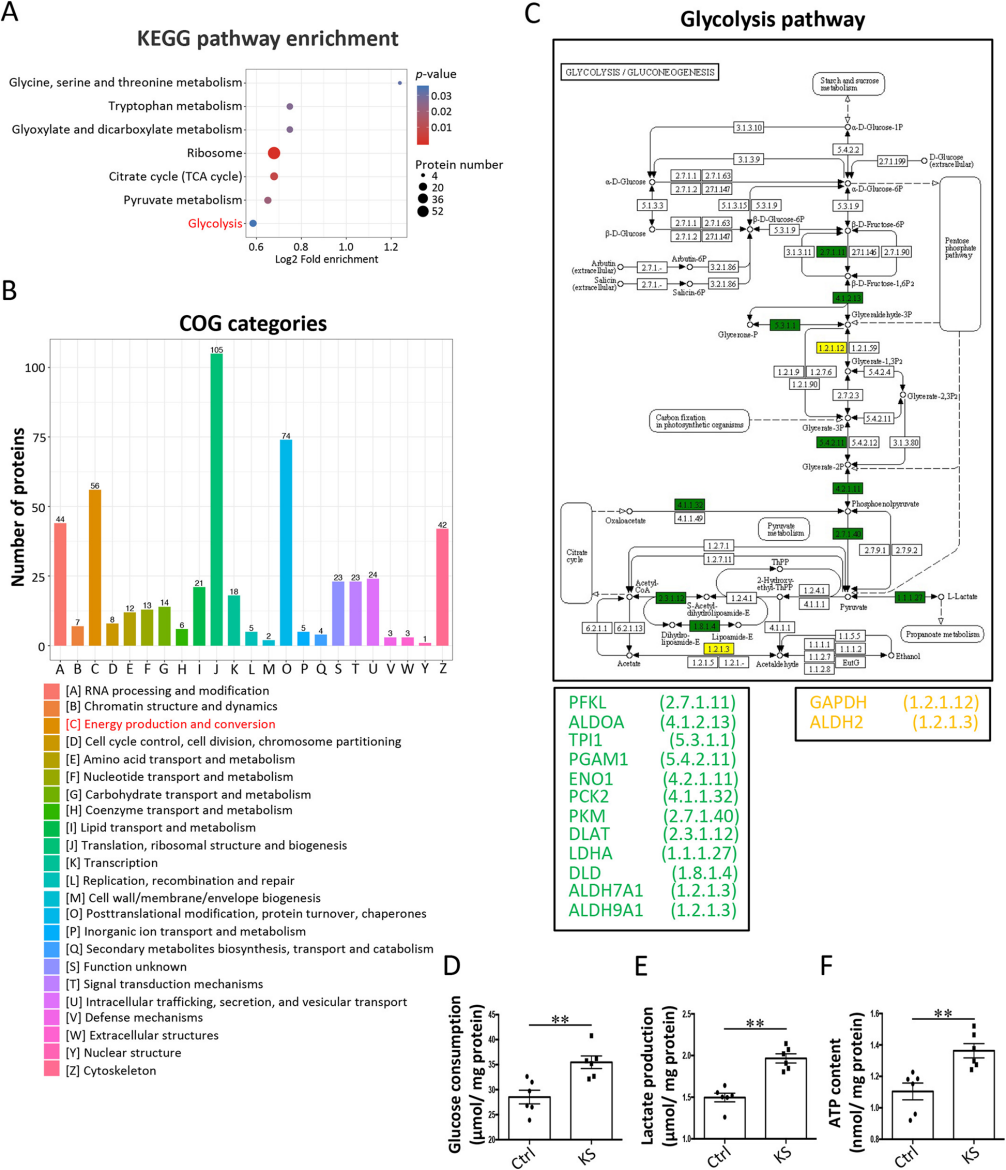
Functional classification and enrichment analyses of differentially lysine-succinylated proteins in IPEC-J2 cells. **A** Enrichment analysis of KEGG pathways by differentially lysine-succinylated proteins. **B** COG annotation of differentially lysine-succinylated proteins. **C** Differentially lysine-succinylated proteins in the glycolysis pathway. The green box shows that the lysine succinylation levels of the corresponding proteins in *K. slooffiae* (KS) group were lower than those in the control (Ctrl) group. The yellow box shows that there were upregulated and downregulated lysine-succinylated sites in the corresponding proteins comparing the KS group with the Ctrl group. **D**–**F** Glucose consumption (**D**), lactate production (**E**), and ATP content (**F**) in IPEC-J2 cells. Data are presented as mean ± SEM (*n* = 6) and evaluated by Student’s *t*-test in **D**–**F**. **p < 0.01

The enrichment analysis of the KEGG pathway revealed that several pathways, including “glycine, serine, and threonine metabolism,” “tryptophan metabolism,” “glyoxylate and dicarboxylate metabolism,” “ribosome,” “citrate cycle,” “pyruvate metabolism,” and “glycolysis” were significantly enriched with differentially lysine-succinylated proteins (Fig. 5A). COG annotation analysis showed that the category “energy production and conversion,” which is associated with glycolysis metabolism, were annotated by differentially lysine-succinylated proteins (Fig. 5B). Enrichment analysis of the data from GO annotation showed that several cellular components, such as the “cytosolic part” that is the location of glycolysis, were significantly enriched with differentially lysine-succinylated proteins (Additional file 7: Fig. S7).

**Fig. 6:**
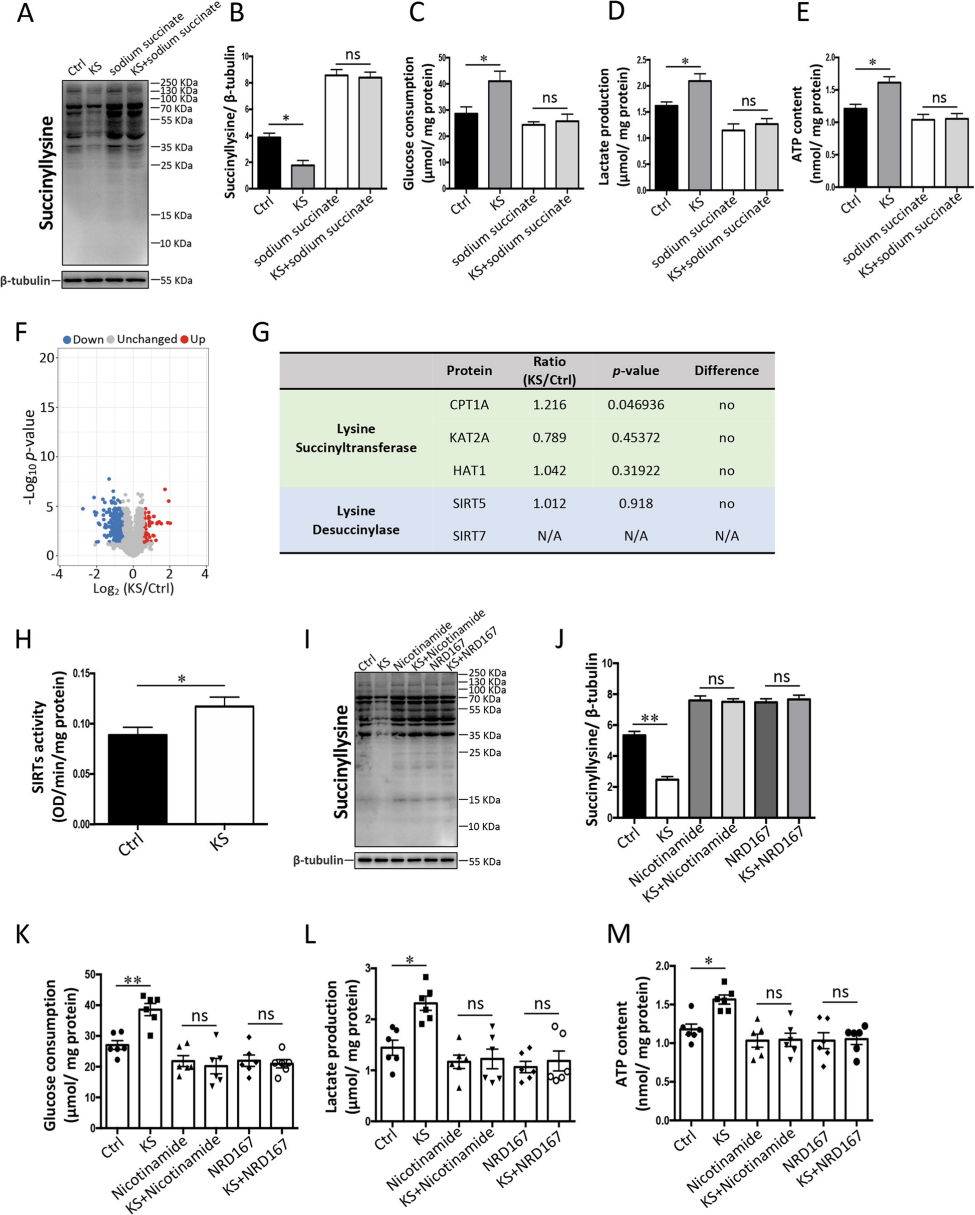
*K. slooffiae* promotes intestinal epithelial glycolysis via lysine desuccinylation induced by SIRT5. **A**, **B** Representative western blots of lysine-succinylated proteins and β-tubulin in IPEC-J2 cells using a pan-lysine succinylation antibody and an anti-β-tubulin antibody, respectively (Ctrl, Control; KS, *K. slooffiae*) (**A**). Quantification of lysine-succinylated proteins levels normalized to β-tubulin levels (**B**). **C**–**E** Glucose consumption (**C**), lactate production (**D**), and ATP content (**E**) in IPEC-J2 cells. **F** Volcano plot analysis of the identified proteins in IPEC-J2 cells. Up (red spots) indicates that the protein levels in KS group were higher than those in the Ctrl group. Down (blue spots) indicates that the protein levels in the KS group were lower than those in the Ctrl group. **G** Analysis of lysine succinyltransferases and lysine desuccinylases in protein levels. **H** Sirtuins (SIRTs) activity in IPEC-J2 cells. **I**, **J** Representative western blots of lysine-succinylated proteins and β-tubulin in IPEC-J2 cells using a pan-lysine succinylation antibody and an anti-β-tubulin antibody, respectively (**I**). Quantification of lysine-succinylated proteins levels normalized to β-tubulin levels (**J**). **K**–**M** Glucose consumption (**K**), lactate production (**L**), and ATP content (**M**) in IPEC-J2 cells. Data are presented as mean ± SEM and were assessed by one-way ANOVA with adjustment for multiple comparisons in **B** (*n* = 3), **C**–**E** (*n* = 6), **J** (*n* = 3), and **K**–**M** (*n* = 6). Data are presented as the mean ± SEM and were evaluated using the Student’s *t*-test in **H** (*n* = 6). **p < 0.01, *p < 0.05; ns, not significant. A ratio of 1.5-fold (>1.50 or <0.67) with a *p*-value < 0.05 was chosen as the cutoff for protein up- or downregulation in **F** (*n* = 3)

Interestingly, the glycolysis pathway was enriched with 59 downregulated lysine-succinylated sites in 14 proteins, and only two upregulated lysine-succinylated sites in two proteins (Fig. 5C), suggesting that the glycolysis pathway may be preferentially affected by *K. slooffiae* treatment. Next, we investigated whether cellular glycolysis metabolism was influenced by *K. slooffiae*. The results showed that *K. slooffiae* significantly increased glucose consumption and lactate production in IPEC-J2 cells (Fig. 5D and E). The levels of ATP in IPEC-J2 cells were also increased with the treatment of *K. slooffiae* (Fig. 5F), indicating an enhanced capacity for energy production. These findings suggested that *K. slooffiae* promoted glycolysis. Furthermore, our data demonstrated that *K. slooffiae*-induced alterations in lysine succinylation levels and glycolysis were blocked by sodium succinate (an activator of lysine succinylation) (Fig. 6A–E), indicating that lysine desuccinylation was essential for *K. slooffiae*-promoted glycolysis.

### The SIRT5-mediated lysine desuccinylation is essential for glycolysis promoted by *K. slooffiae*

To investigate the mechanism of *K. slooffiae*-induced decreases in the lysine succinylation levels, we used a comparative proteomics strategy to analyze the protein expressions affected by *K. slooffiae*. In total, 4849 proteins were quantified using proteomics. Of these, 66 protein levels were increased, and 293 protein levels were decreased by *K. slooffiae* (Fig. 6F). Interestingly, the protein levels of CPT1A, KAT2A, and HAT1, which are lysine succinyltransferases [87–89], were not significantly altered by *K. slooffiae* (Fig. 6G). The protein levels of SIRT5 and SIRT7, which are lysine desuccinylases [90–92], were not significantly altered by *K. slooffiae* (Fig. 6G). However, the total activity of SIRTs was significantly increased by *K. slooffiae* (Fig. 6H). The *K. slooffiae*-induced alterations in lysine succinylation levels and glycolysis were inhibited by nicotinamide (a broad-spectrum sirtuin inhibitor) (Fig. 6I–M). Furthermore, *K. slooffiae*-induced alterations in lysine succinylation levels and glycolysis were inhibited by NRD167 (a SIRT5-specific inhibitor) (Fig. 6I–M). These findings indicated that SIRT5-mediated lysine desuccinylation is essential for glycolysis promoted by *K. slooffiae*.

**Fig. 7:**
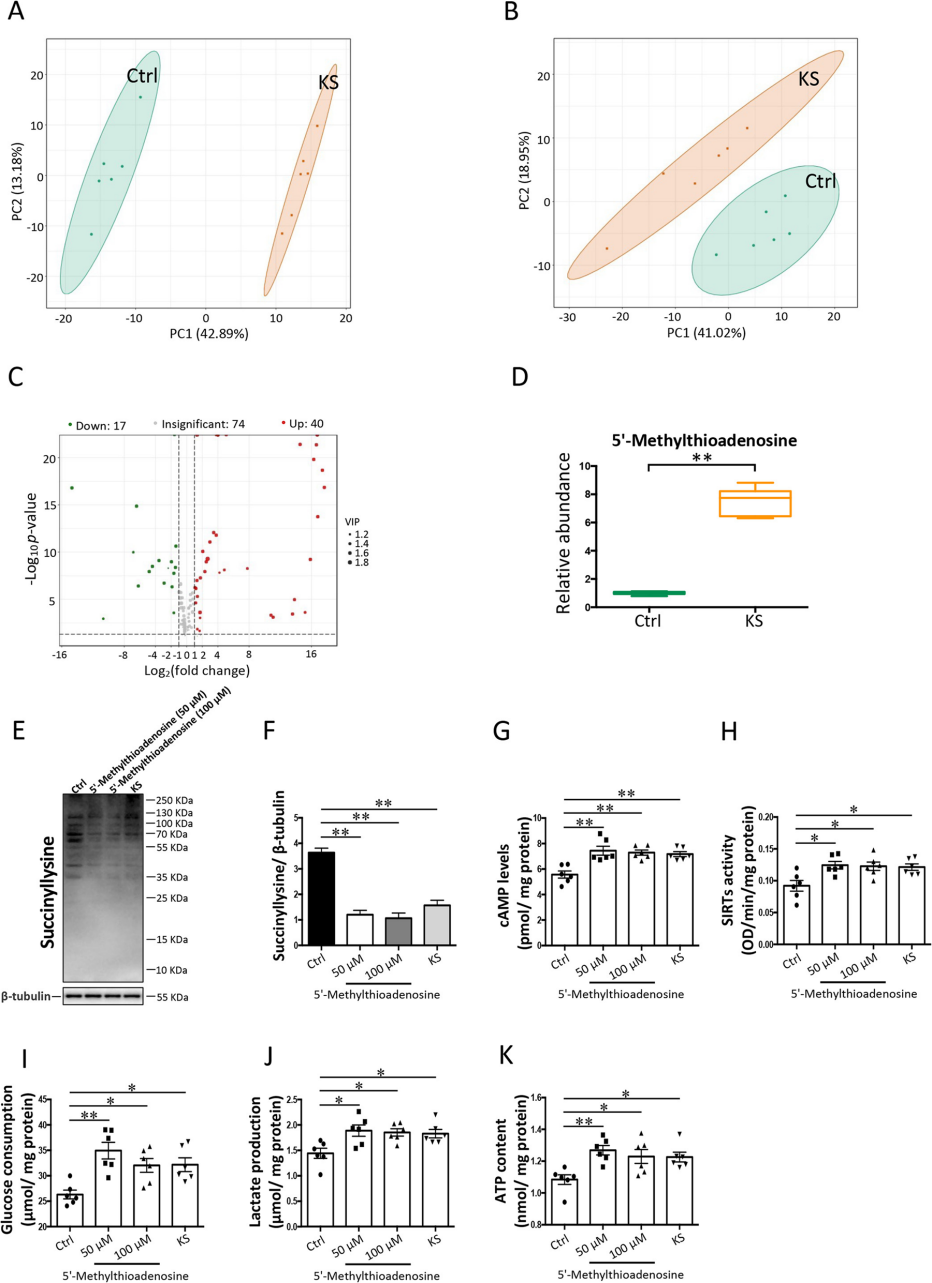
*K. slooffiae*-derived metabolite promotes intestinal epithelial glycolysis mediated by lysine desuccinylation. **A**, **B** PCA of metabolites identified in the culture medium and culture supernatant of *K. slooffiae* in negative ion mode (**A**) and positive ion mode (**B**), respectively (Ctrl, Control; KS, *K. slooffiae*). **C** Volcano plot analysis of all metabolites. **D** Relative abundance analysis of 5′-methylthioadenosine in the culture supernatant of *K. slooffiae*. **E**, **F** Representative western blots of lysine-succinylated proteins and β-tubulin in IPEC-J2 cells using a pan-lysine succinylation antibody and an anti-β-tubulin antibody, respectively (**E**). Quantification of lysine-succinylated proteins levels normalized to β-tubulin levels (**F**). **G** cAMP levels in IPEC-J2 cells. **H** Sirtuins (SIRTs) activity in IPEC-J2 cells. **I**–**K** Glucose consumption (**I**), lactate production (**J**), and ATP content (**K**) in IPEC-J2 cells. The data are presented as the mean ± SEM and evaluated by one-way ANOVA with adjustment for multiple comparisons in **F** (*n* = 3) and **G**–**K** (*n* = 6). Data were assessed by VIP ≥ 1 from the OPLS-DA, absolute Log2 (fold-change) ≥ 1, and *p*-value < 0.05, in **C** and **D** (*n* = 6). **p < 0.01, *p < 0.05

Growing evidence suggests that microbial metabolites may be intermediates in host-gut microbe interactions [93]. Thus, we performed a metabolomic analysis to identify the metabolites produced by *K. slooffiae*. The results of PCA indicated an obvious difference in metabolite composition between control and *K. slooffiae* groups (Fig. 7A and B). Volcano plot further showed that 40 metabolites were produced by *K. slooffiae* (Fig. 7C). Previous studies have revealed that cAMP-PKA signaling contributes to the activation of SIRTs, including SIRT5 [94, 95]. Interestingly, of the 40 metabolites produced by *K. slooffiae*, 5′-methylthioadenosine (Fig. 7D) contributed to the activation of cAMP-PKA signaling, as reported previously [59, 60, 96]. Our results demonstrated that 5′-methylthioadenosine decreased lysine succinylation levels in IPEC-J2 cells (Fig. 7E and F). The cAMP levels and SIRTs activity in IPEC-J2 cells were also increased by 5′-methylthioadenosine treatment (Fig. 7G and H). Glycolysis was promoted by 5′-methylthioadenosine treatment, as evidenced by increased glucose consumption (Fig. 7I), lactate production (Fig. 7J), and ATP levels (Fig. 7K). These findings suggest that *K. slooffiae*-derived 5′-methylthioadenosine promotes SIRT5-mediated lysine desuccinylation, enhancing intestinal epithelial glycolysis.

### *K. slooffiae*-promoted intestinal epithelial glycolysis mediated by lysine desuccinylation is further validated in vivo

**Fig. 8:**
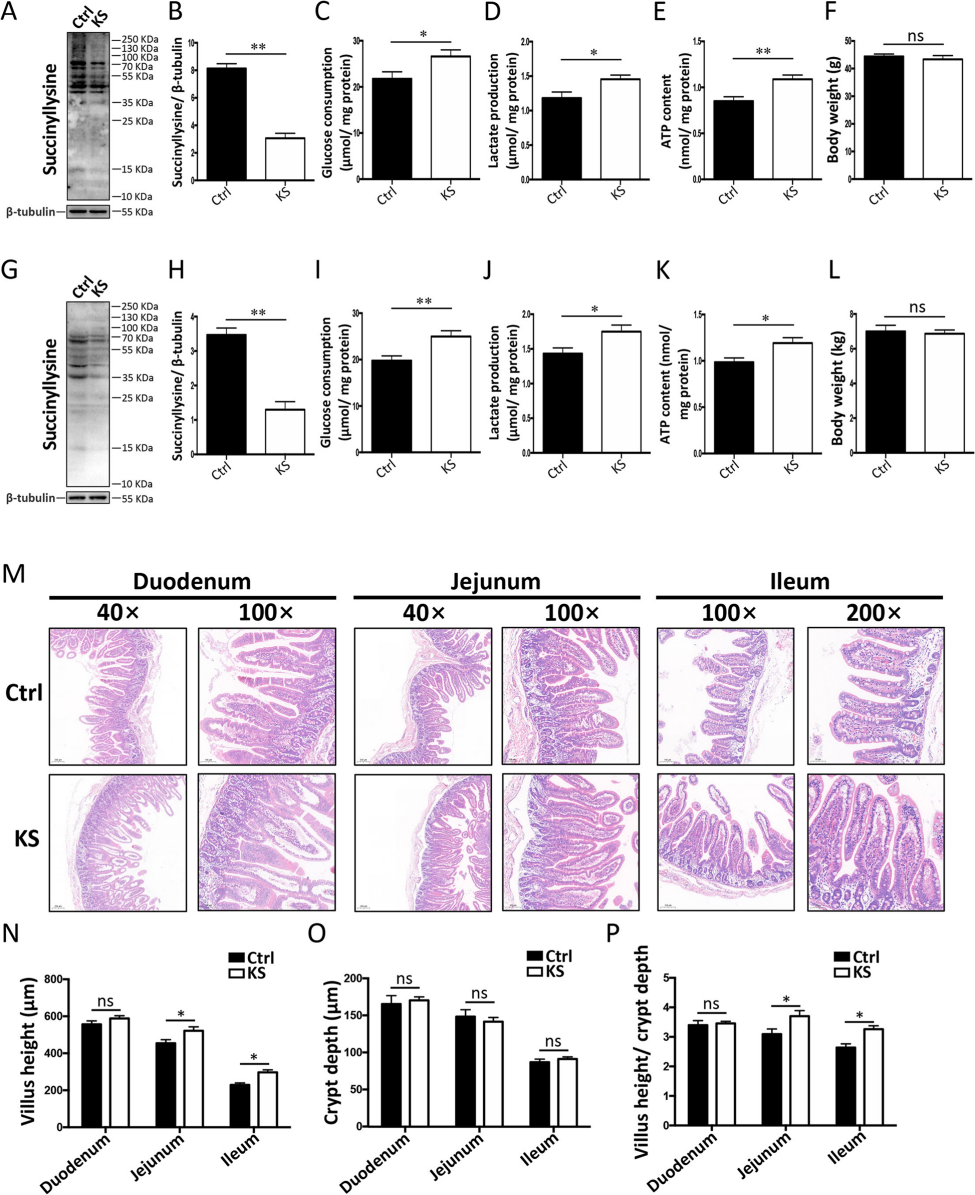
*K. slooffiae* promotes intestinal epithelial glycolysis in vivo. **A**, **B** Representative western blots of lysine-succinylated proteins and β-tubulin in jejunal epithelial cells of GF mice using a pan-lysine succinylation antibody and an anti-β-tubulin antibody, respectively (Ctrl, Control; KS, *K. slooffiae*) (**A**). Quantification of lysine-succinylated proteins levels normalized to β-tubulin levels (**B**). **C**–**E** Glucose consumption (**C**), lactate production (**D**), and ATP content (**E**) in jejunal epithelial cells of GF mice. **F** Body weights of GF mice. **G**, **H** Representative western blots of lysine-succinylated proteins and β-tubulin in jejunal epithelial cells of piglets using a pan-lysine succinylation antibody and an anti-β-tubulin antibody, respectively (**G**). Quantification of lysine-succinylated proteins levels normalized to β-tubulin levels (**H**). **I**–**K** Glucose consumption (**I**), lactate production (**J**), and ATP content (**K**) in the jejunal epithelial cells of piglets. **L** Body weights of piglets. **M** Representative images of intestinal histological morphology by H&E staining. **N**–**P** Statistical analysis of villus height (**N**), crypt depth (**O**), and the ratio of the villus height to crypt depth (**P**). Data are presented as mean ± SEM and evaluated using Student’s *t*-test in **B**–**F** and **H**–**L**. Data are presented as mean ± SEM and evaluated by two-way ANOVA with adjustment for multiple comparisons in **N**–**P**; *n* = 10 (Ctrl group in **C**–**F**), *n* = 8 (KS group in **C**–**F**), *n* = 10 (**I**–**L**), *n* = 5 (**N**–**P**), *n* = 3 (**B** and **H**). **p < 0.01, *p < 0.05, ns, not significant

Next, we evaluated the function of *K. slooffiae* in intestinal epithelial cells of GF mice and pigs. GF mice are free from microbes and have been considered a critical model for investigating the interplay between gut microbiota and host [97–99]. The use of GF mouse model facilitates studying the unique roles of candidate microbes in the host by eliminating the potential effects of other microbes in the host. The results showed that lysine succinylation levels in jejunal epithelial cells of GF mice were higher than those in SPF mice (Additional file 8: Fig. S8), further suggesting the potential role of gut microbiota in lysine succinylation in jejunal epithelial cells. The *K. sloffiae* is not a commensal fungus in mice. Thus, the difference of lysine succinylation between GF and SPF mice cannot be attributed to this fungus. Our results indicated that oral administration of *K. slooffiae* decreased the lysine succinylation in the jejunal epithelial cells of GF mice (Fig. 8A and B). The oral gavage of *K. slooffiae* also increased glucose consumption (Fig. 8C), lactate production (Fig. 8D), and ATP levels (Fig. 8E) in the jejunal epithelial cells of GF mice, suggesting increased glycolysis. The body weights of GF mice were not significantly altered by *K. slooffiae* (Fig. 8F). The results showed that *K. slooffiae* was isolated from the feces and jejunal contents of the GF mice that were orally administrated *K. slooffiae* (Additional file 9: Fig. S9). These results showed that *K. slooffiae* in feces and jejunal contents of GF mice was living, indicating that *K. slooffiae* stably colonized the intestinal tract of mice in our experiments.

Our results revealed that oral administration of *K. slooffiae* decreased the lysine succinylation in jejunal epithelial cells of DLY piglets (Fig. 8G and H). Oral administration of *K. slooffiae* also increased glucose consumption (Fig. 8I), lactate production (Fig. 8J), and ATP levels (Fig. 8K) in the jejunal epithelial cells of DLY piglets, suggesting increased glycolysis. The body weights of DLY pigs were not significantly altered by *K. slooffiae* (Fig. 8L). Intestinal morphology analysis of DLY pigs showed that the villus height and the ratio of villus height to crypt depth in the jejunum and ileum were increased by oral administration of *K. slooffiae*, suggesting an improvement in intestinal epithelial physiological function (Fig. 8M–P).

As the relative abundances of *K. slooffiae* in DLY, SZL, and NX finishing pigs were higher than those in TM, LW, CM, and HM finishing pigs (Fig. 2C), we hypothesized that lysine desuccinylation-mediated glycolysis might differ among pig breeds. Thus, we compared two representative pig breeds (DLY and CM), and the results showed that jejunal epithelial protein lysine succinylation levels were lower in DLY finishing pigs than those in CM finishing pigs (Additional file 10: Fig. S10A and B). Jejunal epithelial glycolysis activity was higher in DLY finishing pigs than those in CM finishing pigs, as evidenced by the higher glucose consumption (Additional file 10: Fig. S10C), lactate production (Additional file 10: Fig. S10D), and ATP production (Additional file 10: Fig. S10E). Thus, these results revealed that *K. slooffiae* decreased lysine succinylation in jejunal epithelial proteins and enhanced the jejunal epithelial glycolysis *in vivo*.

## Discussion

Here, we report that *K. slooffiae*, the most abundant gut fungal species in weaned piglets, promotes intestinal epithelial glycolysis. Mechanistically, *K. slooffiae* promoted intestinal epithelial glycolysis through lysine desuccinylation by activating 5′-methylthioadenosine metabolite-mediated SIRT5 activity. Most previous studies have focused on gut bacterial communities in mammals and linked gut bacteria to host health [13, 100]. However, fungi are increasingly recognized as important member of the gut microbiota and might be involved in the host health [12]. We analyzed the gut mycobiome of seven pig breeds, and our data provide a landscape of pig gut fungal communities in China. Gut fungal diversity in finishing pigs was higher than that in weaned piglets, which is consistent with the fact that gut bacterial diversity increases with the age in mammals [101–103]. A recent study demonstrated that gut fungal diversity remained steady across different sampling time points (1-35 days after birth) in pigs [37]. Another recent study showed a decrease in gut fungal diversity with the sampling time points (1–35 days after birth), and most of the decrease occurred from 21 to 35 days after birth in pigs weaned at 21 days of age [6]. The dynamic inconsistency in gut fungal diversity in pigs may be because the span of days of age for sampling was different between our study and previous studies [6, 37]. Recent studies have studied the links between gut fungal communities and the short-chain fatty acids levels in pigs [41, 42], and the effects of dietary carbohydrate composition on gut fungal communities [104].

In this study, *K. slooffiae* appears to be abundant in all seven breeds of weaned piglets but only in some breeds of finishing pigs. In particular, the relative abundances of *K. slooffiae* in TM, LW, CM, and HM finishing pigs were lower than those in DLY, SZL, and NX finishing pigs. A previous study suggested that the diet rich in cellulose, a kind of crude fiber, had an adverse effect on the growth of intestinal *K. slooffiae*, and thus affected the *K. slooffiae* abundances in pigs [85]. In our study, the level of crude fiber in the diets of TM, LW, CM, and HM finishing pigs was higher than those in the diets of DLY, SZL, and NX finishing pigs (Additional file 12: Table S1-14). This high proportion of crude fiber in the diets of TM, LW, CM, and HM finishing pigs may have adverse effects on the growth of *K. slooffiae* for two possible reasons: (1) crude fibers may not be suitable for *K. slooffiae* growth and (2) feeding diets containing more crude fiber may lead to higher enrichment of gut fiber-degrading bacteria. These fiber-degrading bacteria may produce many antimicrobial substances, such as organic acids, that inhibit the growth of *K. slooffiae*. In addition, antifungal-specific antibodies in maternal milk may also play important roles in gut fungal abundances in offspring as previously described [105], and this potential role may mainly act on the suckling pig. Together, these findings suggested that the relative abundances of *K. slooffiae* in finishing pigs may be affected by the composition of diet.

Our data provide a panoramic view of the gut fungal communities of pigs and suggest that *K. slooffiae* is the most abundant species that dominate the gut fungal communities in weaned piglets. Previous studies have suggested that the gut microbial community results from 16S rDNA/ITS amplicon sequencing and metagenomics may be distinct [106, 107]. In this study, the results of gut fungal communities revealed by ITS gene amplicon sequencing and metagenomics indicate some differences, such as the phylogenetic tree and genus-level taxonomic composition. The phylogeny results from ITS gene amplicon sequencing did not overlap with those from metagenomics, and fewer fungal genera were detected in metagenomics than those in ITS gene amplicon sequencing, as shown in phylogenetic tree. This difference may be due to the lower proportions of fungal genes as compared to that of bacterial genes [108], and thus, the numbers of ITS reads used for species annotation in metagenomics were fewer than those in ITS gene amplicon sequencing. The results of gut microbial community analysis using 16S rDNA/ITS amplicon sequencing may be affected by PCR biases related to amplification and primer mismatch [44]. The genus *Piromyces* was abundant in the results of metagenomics but not annotated in the results of ITS gene amplicon sequencing, and this difference may be caused by PCR biases. However, both fungal ITS gene amplicon sequencing and metagenomics showed that *K. slooffiae* was a core-predominant gut fungal species. Importantly, recent studies have shown temporal dynamics in the gut fungal communities of pigs and have also suggested that *Kazachstania* was the dominant fungal genus and *K. slooffiae* was the dominant fungal species [6, 37, 38, 109]. These findings suggest that *K. slooffiae* is a characteristic gut fungal species in pigs and may play a significant role in host health.

Interestingly, our data demonstrated that the suitable growth temperature of *K. slooffiae* was 36 to 42 °C, whereas the suitable growth temperature of most yeasts, such as *Saccharomyces cerevisiae*, is 25 to 30 °C [110, 111]. Importantly, a recent report studied morphology, growth curves, antifungal susceptibility, phylogenetic analysis, and biofilm assessment of *K. slooffiae* [7]. *K. slooffiae* was cultured at 37 °C in this previous study [7]. These results lay the foundation for understanding why *K. slooffiae* can adapt to live in the gut lumen of pigs and is the most abundant gut fungal species. Thus, *K. slooffiae* might exhibit powerful metabolic activities in the intestinal tract and plays a critical regulatory role in host metabolism. Previous studies have also identified *K. slooffiae* as a member of the intestinal yeasts in pigs [7, 30, 38, 86, 112–117] and have shown that *K. slooffiae* can produce some bioactive substances, such as peptides, formic acid, and dehydroascorbic acid [118]. The total nitrogen and amino acid contents of *K. slooffiae* were higher than those of *S. cerevisiae* [118]. Thus, these findings suggest a unique characteristic of *K. slooffiae* and that the gut lumen of pigs is suitable for its growth.

Growing evidence has linked gut fungal dysbiosis to host diseases, including CRC, primary sclerosing cholangitis, and IBD [8–10]. Intestinal fungi have critical functions in the activation of innate immune response in host [119, 120]. Recent studies showed that Candidalysin, a pore-forming peptide toxin secreted by *Candida albicans*, is a crucial virulence factor. *C. albicans*-derived candidalysin activates the MAPK-cFos-MPK1 pathway and increases the production of pro-inflammatory cytokines, thereby activating the innate immune response [119, 120]. Furthermore, gut fungal species, including *C. albicans* and *S. cerevisiae*, can activate host adaptive immune responses, such as the Th17 immune response [11, 121]. Gut fungi also confer protection against intestinal infections and inflammatory disorders, consistent with the functions of enteric bacteria [122]. We demonstrated that *K. slooffiae* promoted intestinal epithelial glycolysis, increasing ATP production. An increased glycolysis rate in healthy epithelial cells may be associated with an enhanced capacity for energy production and consequently improvement of basic intestinal functions, such as motility, absorption, and secretion. Intestinal epithelial oxidative phosphorylation was activated by *Lactobacillus gasseri* LA39 [123]. These findings reveal an important contribution of gut microbiota to host energy metabolism, further suggesting a promising avenue of manipulating intestinal microbiota to prevent insufficient energy supply induced by gastrointestinal disorders.

Our study revealed that *K. slooffiae*-driven intestinal glycolysis is mediated by lysine desuccinylation. Previous studies have shown that the protein expression [19, 20], as well as the protein PTMs, such as phosphorylation and crotonylation [21–23], in host cells can be mediated by microbes, including viruses and bacteria. Some bacterial toxin effectors influence the host through PTM regulations, such as ubiquitination [24], glycosylation [26, 124], and fatty acylation [28, 125]. Gut microbiota-derived metabolites, such as inositol phosphate and short-chain fatty acids, play critical regulatory roles in host PTMs, such as crotonylation and acetylation [21, 126]. However, it is unclear whether gut fungi induce alterations in host protein PTMs, and the underlying mechanisms have not yet been elucidated. Our results demonstrated that *K. slooffiae* decreased the intestinal epithelial lysine succinylation, resulting in enhanced glycolysis. Furthermore, we found that SIRT5-mediated lysine desuccinylation was essential for glycolysis promoted by *K. slooffiae*. Thus, our findings provide a new perspective on gut microbiota-mediated host PTM regulation and the mechanisms of host–gut fungi interactions.

## Conclusions

In sum, we analyzed the pig gut fungal communities and showed that *K. slooffiae*, the most abundant gut fungal species, promoted intestinal epithelial glycolysis through lysine desuccinylation by activating SIRT5. Our data indicate that gut fungi play important roles in host metabolism by regulating host PTMs. These findings suggest a potential protective avenue for pigs with an insufficient intestinal energy supply.

## Supplementary Information

**Additional file 1: Fig. S1.** Geographic distribution of fecal samples and rarefaction curves analysis of fungal ITS gene amplicon sequencing. A, Geographic distribution of fecal samples from pigs in China (DLY, Duroc × [Landrace × Yorkshire]; TM, Tibetan miniature; LW, Laiwu; SZL, Shaziling; CM, Congjiang miniature; HM, Huanjiang miniature; and NX, Ningxiang). B, Rarefaction curves based on fungal Chao index.

**Additional file 2: Fig. S2.** Species-level taxonomic composition and phylogenetic tree analysis of gut fungal communities in pigs revealed by ITS gene amplicon sequencing. A, Gut fungal taxonomic composition at the species level in 112 pigs (DLY, Duroc × [Landrace × Yorkshire]; TM, Tibetan miniature; LW, Laiwu; SZL, Shaziling; CM, Congjiang miniature; HM, Huanjiang miniature; and NX, Ningxiang). The results are shown according to the samples. B, Phylogenetic tree analysis of gut fungal genera in pigs.

**Additional file 3: Fig. S3.** Gene length distribution of gene catalog and phylogenetic tree analysis of gut fungal genera in pigs revealed by metagenomics. A, Gene length distribution of gene catalog in gut microbiome of pigs. B, Gut fungal phylogenetic tree analysis of pigs revealed by metagenomics. Gut fungal genera belonging to the same phyla are marked by the same color, whereas those gut fungal genera belonging to different phyla are marked by different colors.

**Additional file 4: Fig. S4.** Co-culture of *K. slooffiae* and IPEC-J2 cells. *K. slooffiae* was added to the cell medium for 3 h or 6 h. The initial proportion of *K. slooffiae* numbers to IPEC-J2 cells numbers was 5:1 or 50:1. Representative pictures for the co-culture of K. slooffiae and IPEC-J2 cells were obtained using a light microscope (Ctrl, Control).

**Additional file 5: Fig. S5.** Analysis of protein PTM levels in IPEC-J2 cells. A-D, Representative western blots of the lysine-crotonylated proteins (A), lysine-2-hydroxyisobutyrylated proteins (B), lysine-acetylated proteins (C), ubiquitinated proteins (D), and β-tubulin in IPEC-J2 cells using the corresponding pan monoclonal antibodies and an anti-β-tubulin antibody, respectively. Western blotting data are representative of at least three independent experiments. E, Analysis of relative standard deviation (RSD) in lysine succinylome.

**Additional file 6: Fig. S6.** Motif enrichment analysis of the succinylome. Detailed information for the motif enrichment analysis using motif-x, included motif logo, motif, motif score, matches, size, and fold increase.

**Additional file 7: Fig. S7.** GO enrichment analysis of differentially lysine-succinylated proteins in IPEC-J2 cells. A-C, Enrichment analyses of cellular components (A), biological processes (B), and molecular functions (C) by differentially lysine-succinylated proteins are shown in bubble charts.

**Additional file 8: Fig. S8.** Comparison of lysine succinylation levels between the SPF and GF mice. A and B, Representative western blots of lysine-succinylated proteins and β-tubulin in jejunal epithelial cells of SPF and GF mice using a pan-lysine succinylation antibody and an anti-β-tubulin antibody, respectively (A). Quantification of lysine-succinylated proteins levels normalized to β-tubulin levels (B).

**Additional file 9: Fig. S9.** Isolation and identification of *K. slooffiae* from the jejunal contents and feces of GF mice gavaged with *K. slooffiae*. A and B, Representative picture for the single colonies grown on the YPD agar plates that were spread with jejunal contents suspension of GF mice (Ctrl, control) (A). Representative BLAST results of the ITS-2 region of the fungal ITS gene used for identification (B). C and D, Representative picture for the single colonies grown on YPD agar plates that were spread with fecal suspension of GF mice (C). Representative BLAST results of the ITS-2 region of the fungal ITS gene used for identification (D).

**Additional file 10: Fig. S10.** Comparison of lysine succinylation levels and glycolysis between the two pig breeds. A and B, Representative western blots of lysine-succinylated proteins and β-tubulin in jejunal epithelial cells of Congjiang miniature (CM) and Duroc × [Landrace × Yorkshire] (DLY) finishing pigs using a pan-lysine succinylation antibody and an anti-β-tubulin antibody, respectively (A). Quantification of lysine-succinylated proteins levels normalized to β-tubulin levels (B). C-E, Glucose consumption (C), lactate production (D), and ATP content (E) in jejunal epithelial cells of CM and DLY finishing pigs. Data are presented as the mean ± SEM and evaluated using Student's t-test in B (n = 3) and C-E (n = 5). **p < 0.01, *p < 0.05.

**Additional file 11: Data S1.** Detailed information on the age and gender of pigs.

**Additional file 12: Table S1.** Composition of diets for DLY weaned piglets. **Table S2.** Composition of diets for TM weaned piglets. **Table S3.** Composition of diets for LW weaned piglets. **Table S4.** Composition of diets for SZL weaned piglets. **Table S5.** Composition of diets for CM weaned piglets. **Table S6.** Composition of diets for HM weaned piglets. **Table S7.** Composition of diets for NX weaned piglets. **Table S8.** Composition of diets for DLY finishing pigs. **Table S9.** Composition of diets for TM finishing pigs. **Table S10.** Composition of diets for LW finishing pigs. **Table S11.** Composition of diets for SZL finishing pigs. **Table S12.** Composition of diets for CM finishing pigs. **Table S13.** Composition of diets for HM finishing pigs. **Table S14.** Composition of diets for NX finishing pigs.

## Data Availability

The raw sequencing data have been deposited in the China National GeneBank Sequence Archive (CNSA) of the China National GeneBank DataBase (CNGBdb) with the accession number CNP0002106 (the review link: http://db.cngb.org/cnsa/project/CNP0002106_fb54395e/reviewlink/).
