# Supplementary figures and images for: Core-predominant gut fungus Kazachstania slooffiae promotes intestinal epithelial glycolysis via lysine desuccinylation in pigs
Source: Microbiome. 2023 Feb 23;11:31. doi: 10.1186/s40168-023-01468-3 (PMC9948344; doi:10.1186/s40168-023-01468-3)

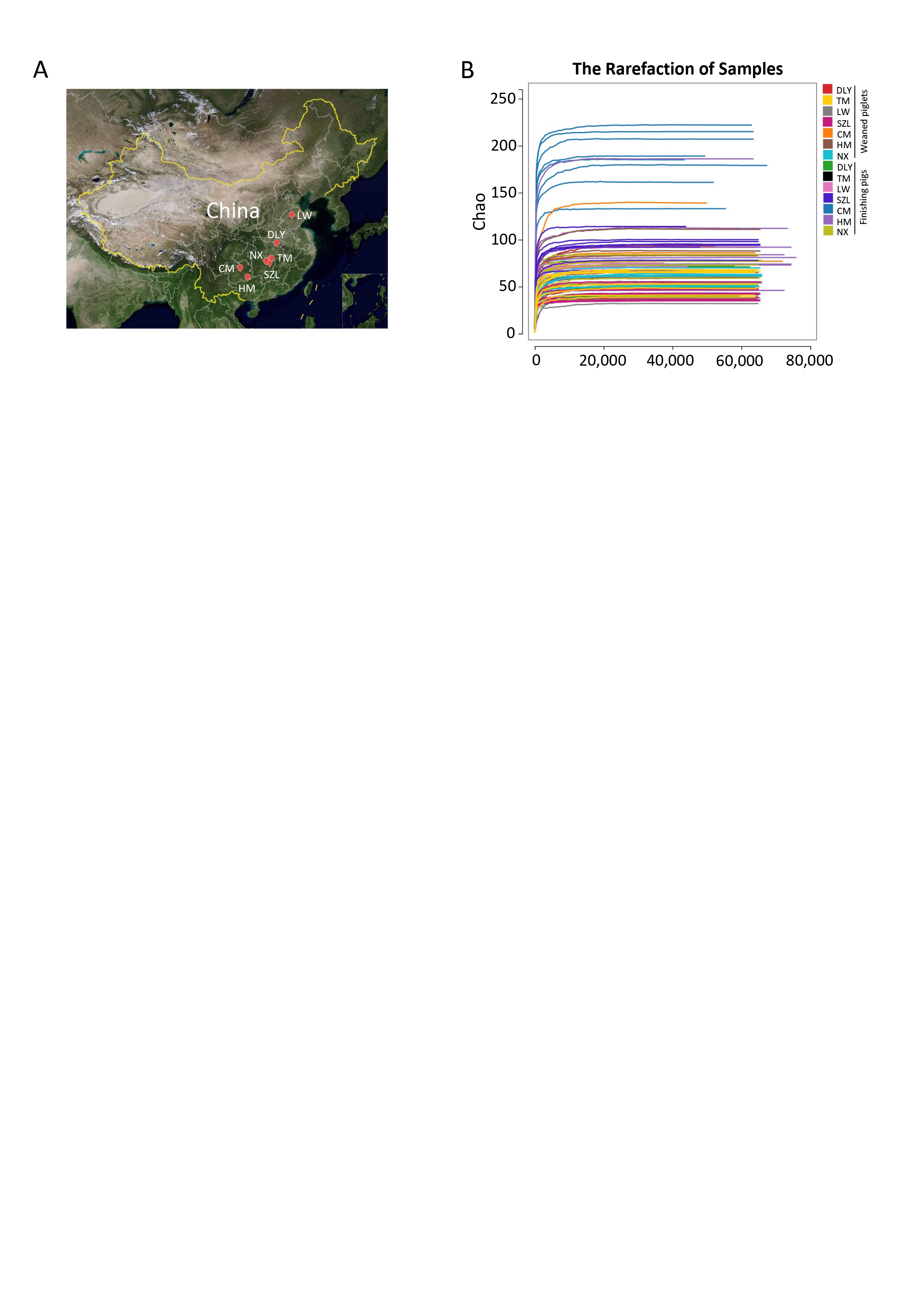

Supplement: Supplementary file 2 — Additional file 1: Fig. S1. Geographic distribution of fecal samples and rarefaction curves analysis of fungal ITS gene amplicon sequencing. A, Geographic distribution of fecal samples from pigs in China (DLY, Duroc × [Landrace × Yorkshire]; TM, Tibetan miniature; LW, Laiwu; SZL, Shaziling; CM, Congjiang miniature; HM, Huanjiang miniature; and NX, Ningxiang). B, Rarefaction curves based on fungal Chao index. [file 40168_2023_1468_MOESM1_ESM.jpg]

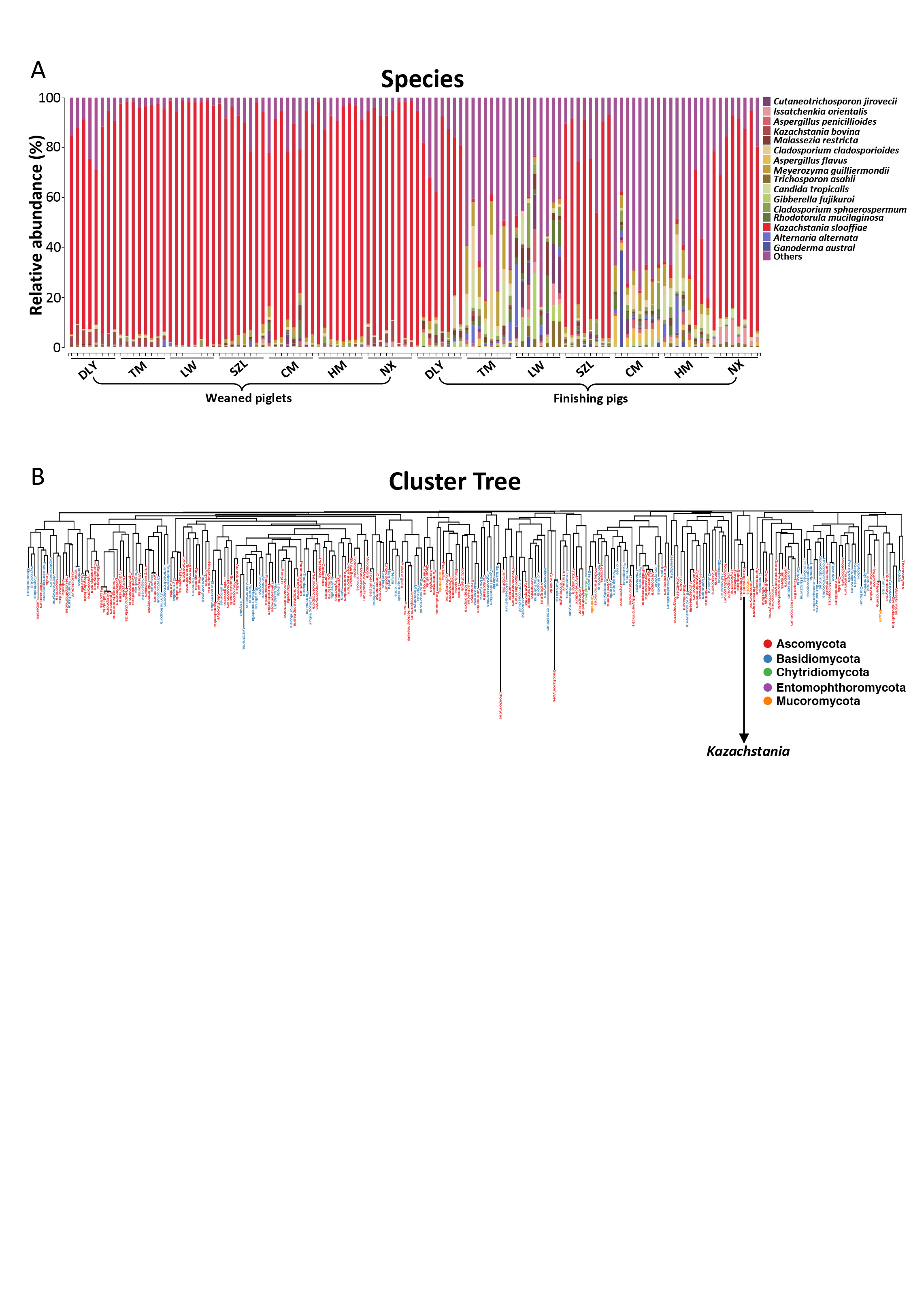

Supplement: Supplementary file 3 — Additional file 2: Fig. S2. Species-level taxonomic composition and phylogenetic tree analysis of gut fungal communities in pigs revealed by ITS gene amplicon sequencing. A, Gut fungal taxonomic composition at the species level in 112 pigs (DLY, Duroc × [Landrace × Yorkshire]; TM, Tibetan miniature; LW, Laiwu; SZL, Shaziling; CM, Congjiang miniature; HM, Huanjiang miniature; and NX, Ningxiang). The results are shown according to the samples. B, Phylogenetic tree analysis of gut fungal genera in pigs. [file 40168_2023_1468_MOESM2_ESM.jpg]

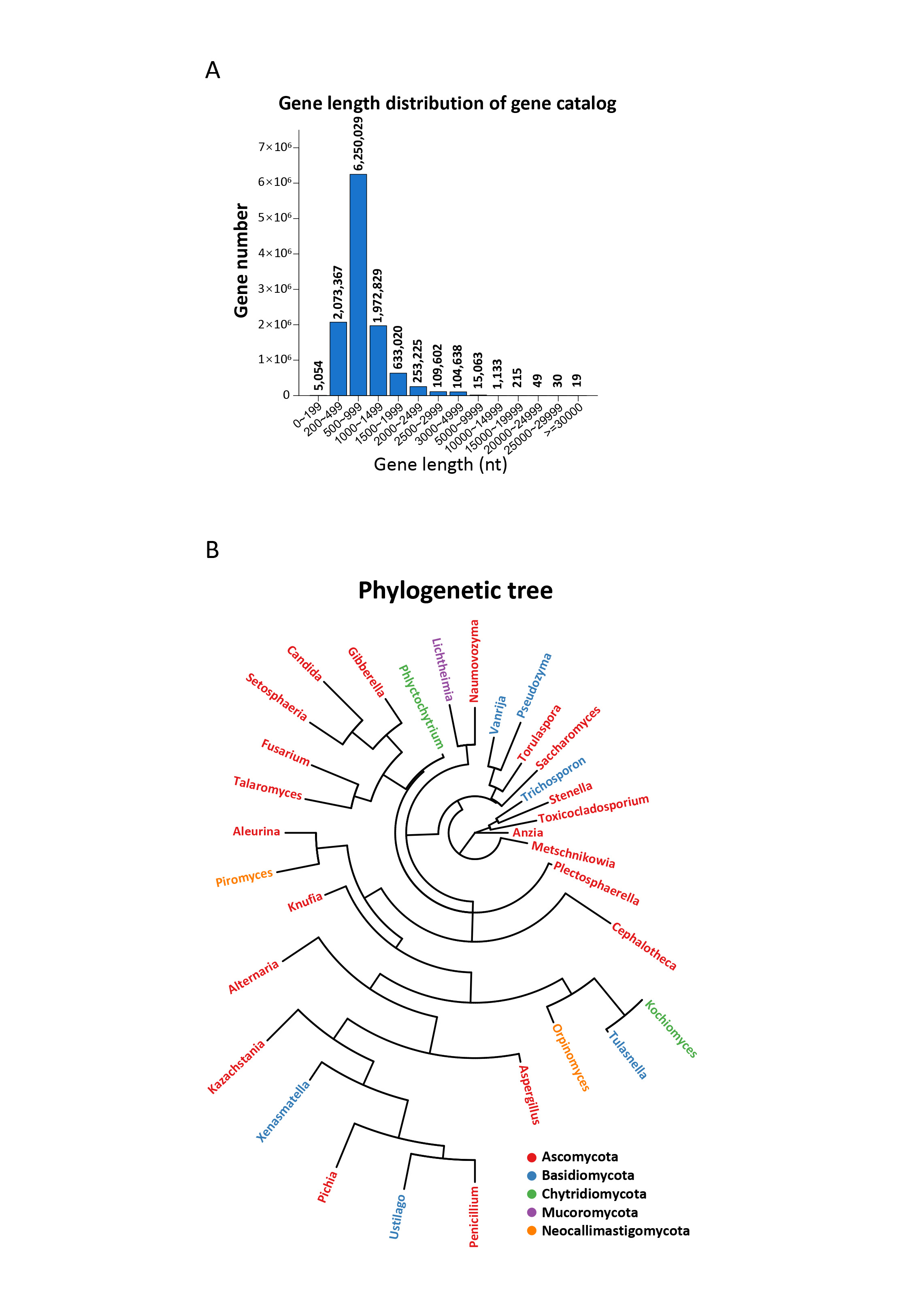

Supplement: Supplementary file 4 — Additional file 3: Fig. S3. Gene length distribution of gene catalog and phylogenetic tree analysis of gut fungal genera in pigs revealed by metagenomics. A, Gene length distribution of gene catalog in gut microbiome of pigs. B, Gut fungal phylogenetic tree analysis of pigs revealed by metagenomics. Gut fungal genera belonging to the same phyla are marked by the same color, whereas those gut fungal genera belonging to different phyla are marked by different colors. [file 40168_2023_1468_MOESM3_ESM.jpg]

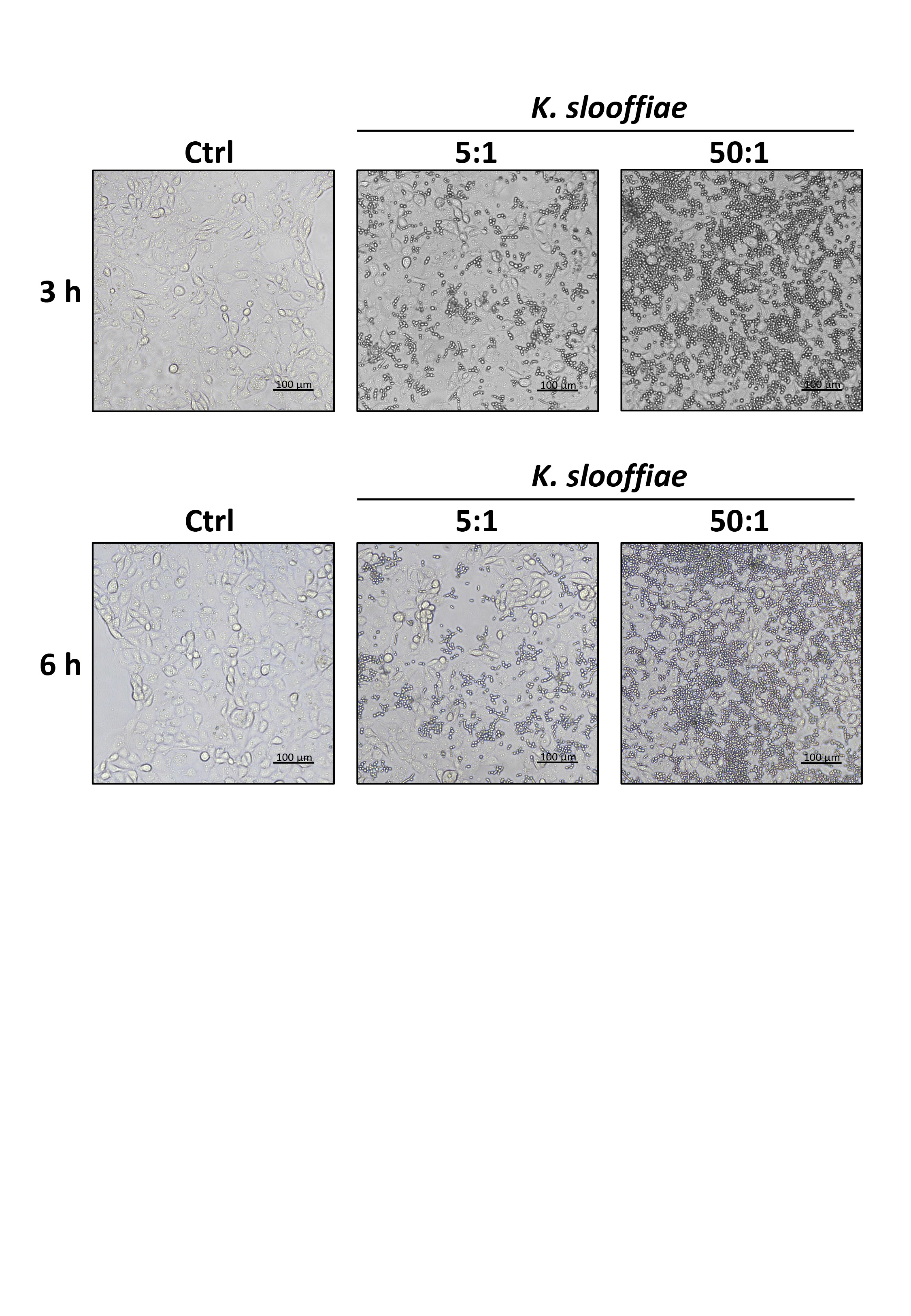

Supplement: Supplementary file 5 — Additional file 4: Fig. S4. Co-culture of K. slooffiae and IPEC-J2 cells. K. slooffiae was added to the cell medium for 3 h or 6 h. The initial proportion of K. slooffiae numbers to IPEC-J2 cells numbers was 5:1 or 50:1. Representative pictures for the co-culture of K. slooffiae and IPEC-J2 cells were obtained using a light microscope (Ctrl, Control). [file 40168_2023_1468_MOESM4_ESM.jpg]

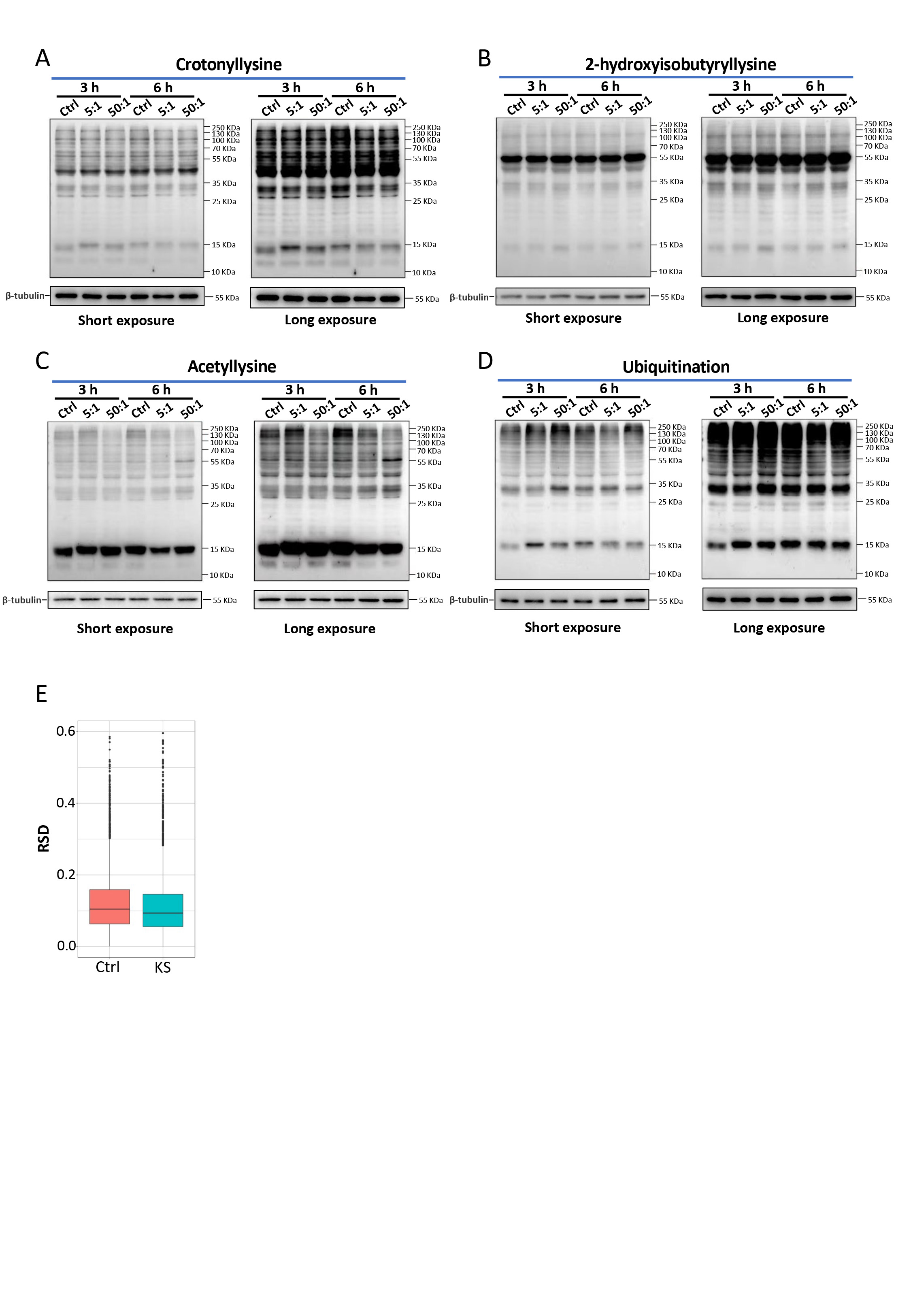

Supplement: Supplementary file 6 — Additional file 5: Fig. S5. Analysis of protein PTM levels in IPEC-J2 cells. A-D, Representative western blots of the lysine-crotonylated proteins (A), lysine-2-hydroxyisobutyrylated proteins (B), lysine-acetylated proteins (C), ubiquitinated proteins (D), and β-tubulin in IPEC-J2 cells using the corresponding pan monoclonal antibodies and an anti-β-tubulin antibody, respectively. Western blotting data are representative of at least three independent experiments. E, Analysis of relative standard deviation (RSD) in lysine succinylome. [file 40168_2023_1468_MOESM5_ESM.jpg]

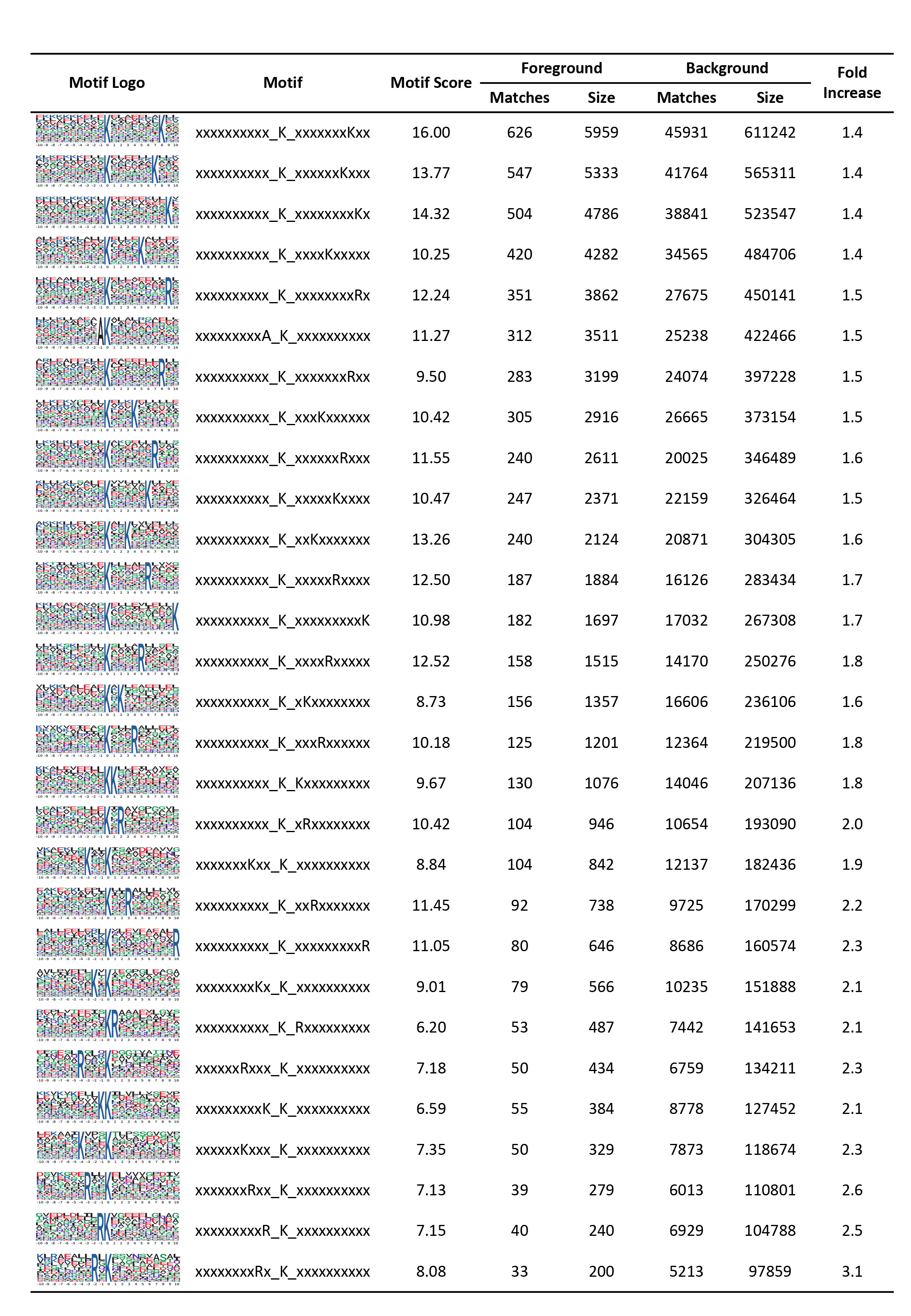

Supplement: Supplementary file 7 — Additional file 6: Fig. S6. Motif enrichment analysis of the succinylome. Detailed information for the motif enrichment analysis using motif-x, included motif logo, motif, motif score, matches, size, and fold increase. [file 40168_2023_1468_MOESM6_ESM.jpg]

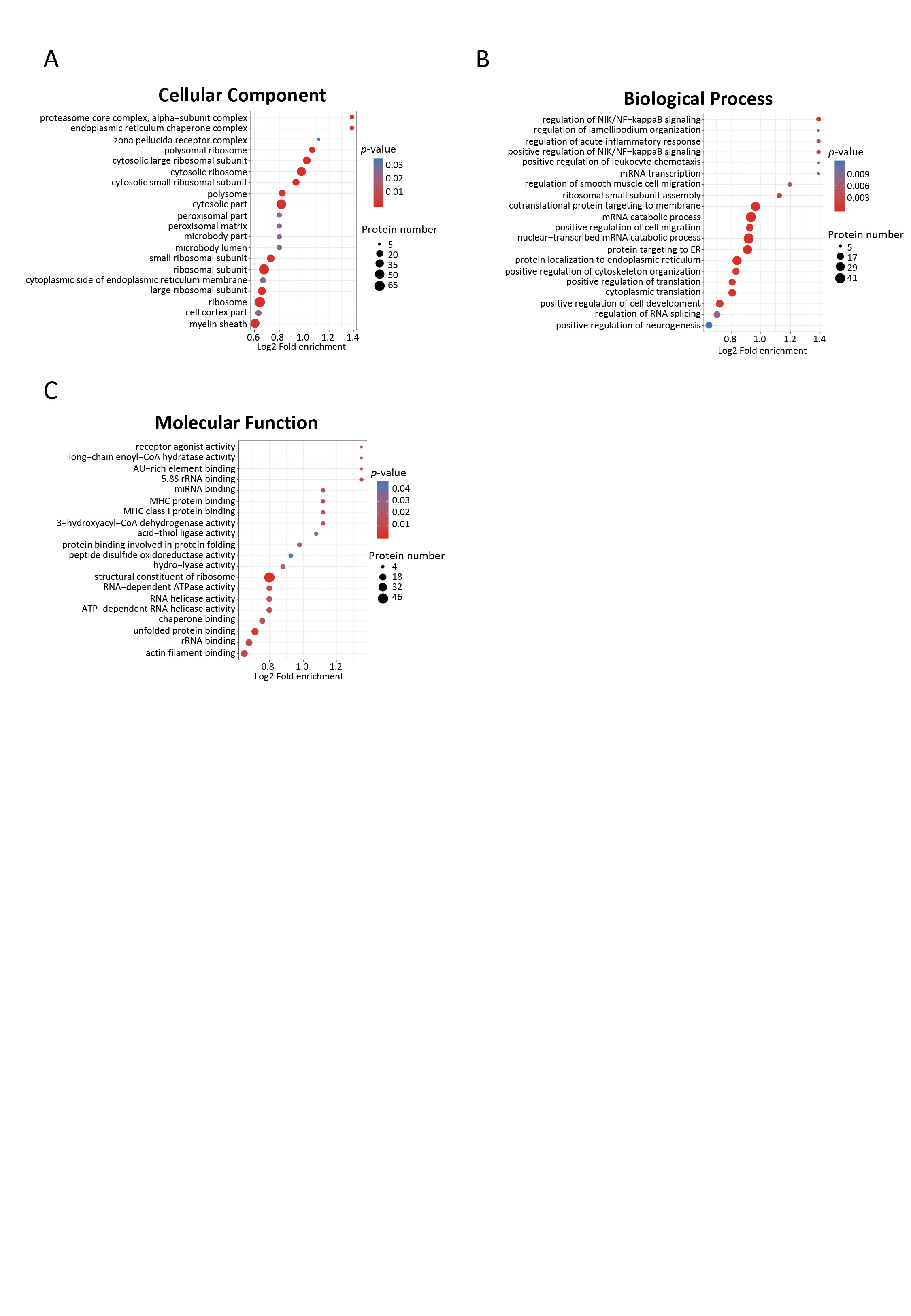

Supplement: Supplementary file 8 — Additional file 7: Fig. S7. GO enrichment analysis of differentially lysine-succinylated proteins in IPEC-J2 cells. A-C, Enrichment analyses of cellular components (A), biological processes (B), and molecular functions (C) by differentially lysine-succinylated proteins are shown in bubble charts. [file 40168_2023_1468_MOESM7_ESM.jpg]

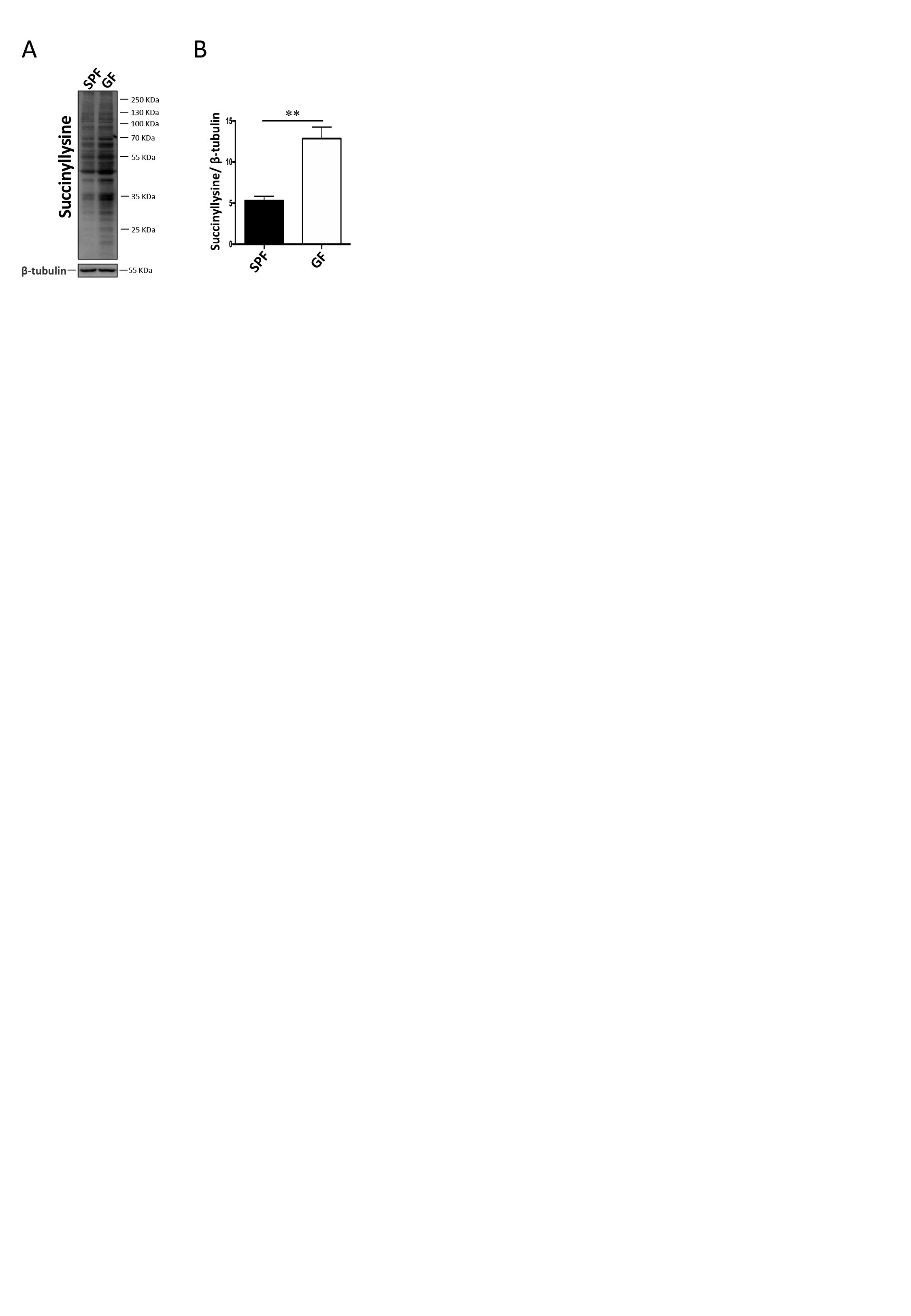

Supplement: Supplementary file 9 — Additional file 8: Fig. S8. Comparison of lysine succinylation levels between the SPF and GF mice. A and B, Representative western blots of lysine-succinylated proteins and β-tubulin in jejunal epithelial cells of SPF and GF mice using a pan-lysine succinylation antibody and an anti-β-tubulin antibody, respectively (A). Quantification of lysine-succinylated proteins levels normalized to β-tubulin levels (B). [file 40168_2023_1468_MOESM8_ESM.jpg]

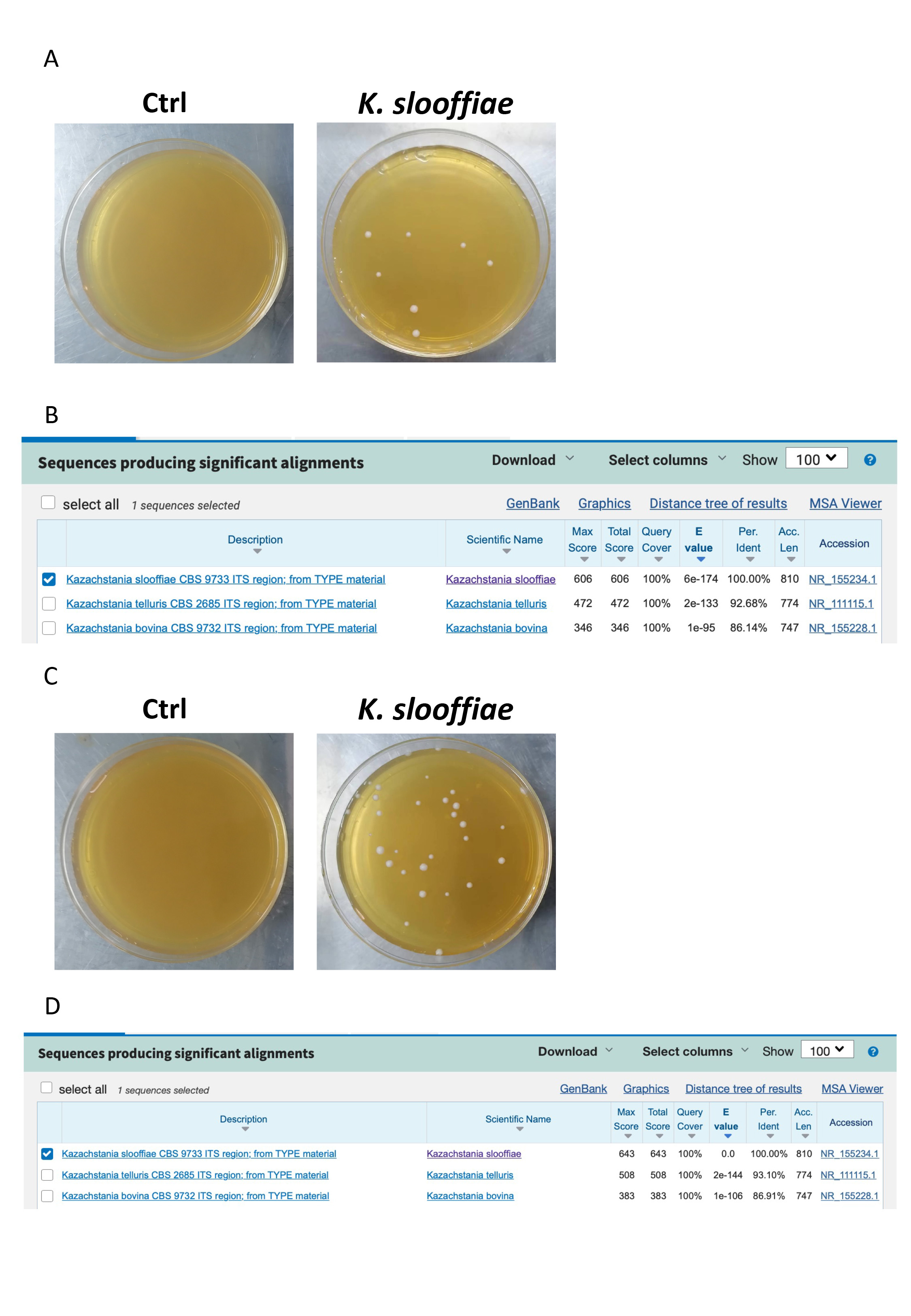

Supplement: Supplementary file 10 — Additional file 9: Fig. S9. Isolation and identification of K. slooffiae from the jejunal contents and feces of GF mice gavaged with K. slooffiae. A and B, Representative picture for the single colonies grown on the YPD agar plates that were spread with jejunal contents suspension of GF mice (Ctrl, control) (A). Representative BLAST results of the ITS-2 region of the fungal ITS gene used for identification (B). C and D, Representative picture for the single colonies grown on YPD agar plates that were spread with fecal suspension of GF mice (C). Representative BLAST results of the ITS-2 region of the fungal ITS gene used for identification (D). [file 40168_2023_1468_MOESM9_ESM.jpg]

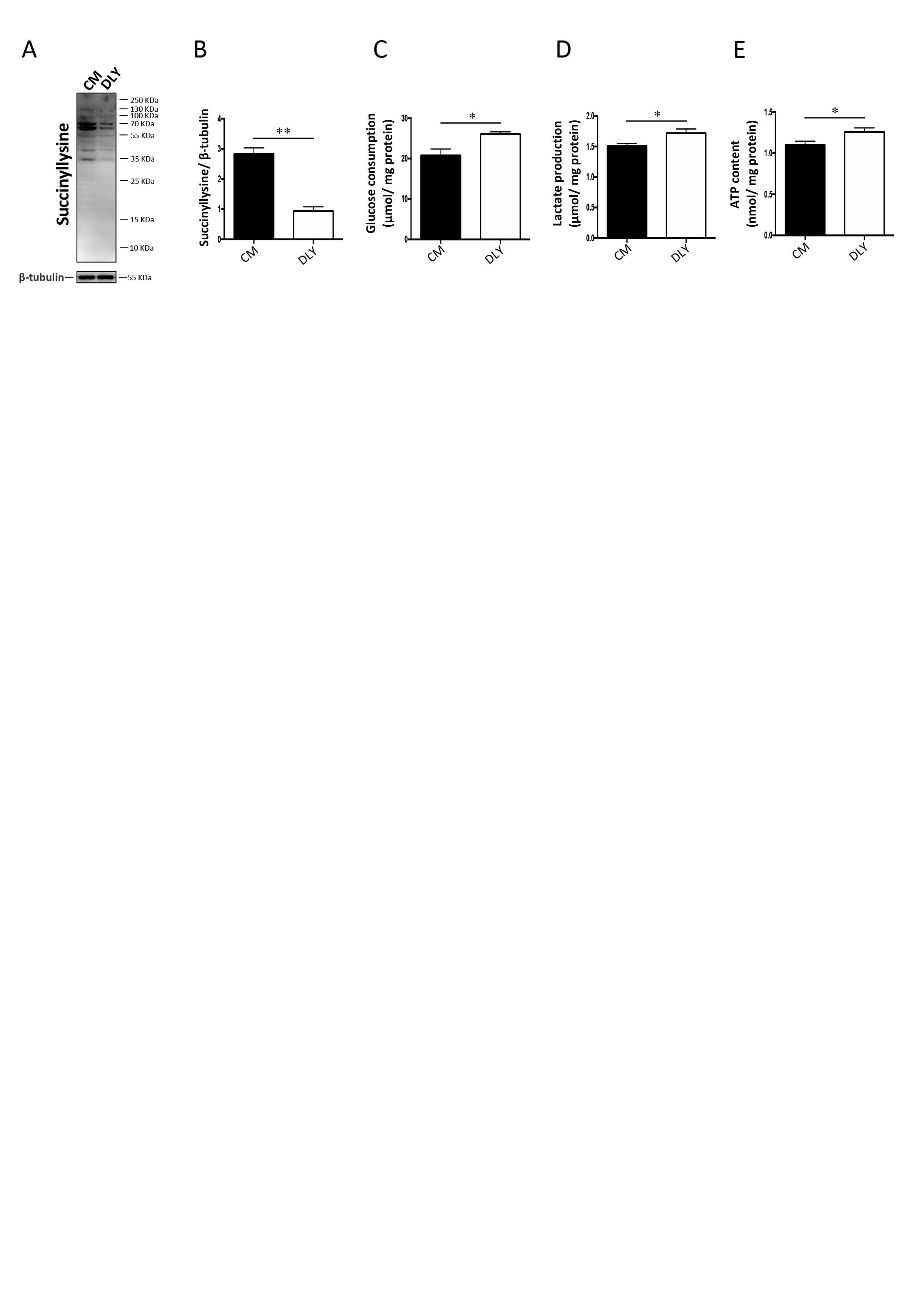

Supplement: Supplementary file 11 — Additional file 10: Fig. S10. Comparison of lysine succinylation levels and glycolysis between the two pig breeds. A and B, Representative western blots of lysine-succinylated proteins and β-tubulin in jejunal epithelial cells of Congjiang miniature (CM) and Duroc × [Landrace × Yorkshire] (DLY) finishing pigs using a pan-lysine succinylation antibody and an anti-β-tubulin antibody, respectively (A). Quantification of lysine-succinylated proteins levels normalized to β-tubulin levels (B). C-E, Glucose consumption (C), lactate production (D), and ATP content (E) in jejunal epithelial cells of CM and DLY finishing pigs. Data are presented as the mean ± SEM and evaluated using Student's t-test in B (n = 3) and C-E (n = 5). **p < 0.01, *p < 0.05. [file 40168_2023_1468_MOESM10_ESM.jpg]
