# Supplementary material for: Core-predominant gut fungus Kazachstania slooffiae promotes intestinal epithelial glycolysis via lysine desuccinylation in pigs
Source: Microbiome. 2023 Feb 23;11:31. doi: 10.1186/s40168-023-01468-3 (PMC9948344; doi:10.1186/s40168-023-01468-3)
Supplement: Supplementary file 12 — Additional file 11: Data S1. Detailed information on the age and gender of pigs. [file 40168_2023_1468_MOESM11_ESM.pdf]

Data S1 The detailed information for age and gender of pigs

Weaned piglets

**Breed**	DLY	TM	LW	SZL	CM	HM	NX
**Age**	35 days	74 days	59 days	49 days	54 days	44 days	49 days
**Gender**	castrated male	castrated male	castrated male	castrated male	castrated male	castrated male	castrated male

Finishing pigs

**Breed**	DLY	TM	LW	SZL	CM	HM	NX
**Age**	6 months	10 months	10 months	8 months	12 months	7 months	8 months
**Gender**	castrated male	castrated male	castrated male	castrated male	castrated male	castrated male	castrated male
