# Supplementary material for: Core-predominant gut fungus Kazachstania slooffiae promotes intestinal epithelial glycolysis via lysine desuccinylation in pigs
Source: Microbiome. 2023 Feb 23;11:31. doi: 10.1186/s40168-023-01468-3 (PMC9948344; doi:10.1186/s40168-023-01468-3)
Supplement: Supplementary file 13 — Additional file 12: Table S1. Composition of diets for DLY weaned piglets. Table S2. Composition of diets for TM weaned piglets. Table S3. Composition of diets for LW weaned piglets. Table S4. Composition of diets for SZL weaned piglets. Table S5. Composition of diets for CM weaned piglets. Table S6. Composition of diets for HM weaned piglets. Table S7. Composition of diets for NX weaned piglets. Table S8. Composition of diets for DLY finishing pigs. Table S9. Composition of diets for TM finishing pigs. Table S10. Composition of diets for LW finishing pigs. Table S11. Composition of diets for SZL finishing pigs. Table S12. Composition of diets for CM finishing pigs. Table S13. Composition of diets for HM finishing pigs. Table S14. Composition of diets for NX finishing pigs. [file 40168_2023_1468_MOESM12_ESM.pdf]

Table S1. Composition of diets for DLY weaned piglets

Ingredient	Content (%)
Corn	55
Soybean meal	25
Fish meal	5
Whey powder	5
Soybean oil	3
Glucose	3
Premix	4
Total	100
Calculated nutrient content	
Dry matter (%)	88.50
Crude protein (%)	19.79
Ether extract (%)	5.86
Crude fiber (%)	2.76
Calcium (%)	0.76
Available phosphorus (%)	0.37
Digestible energy (MJ/kg)	14.47

The Premix provided the following nutrients per kilogram of diet: 3.0 g L-lysine HCl, 1.0 g DL-methionine, 1.0 g L-threonine, 0.1 g L-tryptophan, 4.08 g calcium, 1.37 g phosphorus, 0.4 g Na, 0.6 g Cl, 90 mg Fe, 10 mg Cu, 10 mg Mn, 90 mg Zn, 0.15 mg I, 0.3 mg Se, 10,000 IU vitamin A, 1,500 IU vitamin D3, 40 IU vitamin E, 2 mg menadione, 3 mg thiamin, 5 mg riboflavin, 30 mg niacin, 20 mg pantothenic acid, 5 mg pyridoxine, 0.15 mg biotin, 1 mg folic acid, 40 μg vitamin B12, and 1.0 g choline.

Table S2. Composition of diets for TM weaned piglets

Ingredient	Content (%)
Corn	60.5
Soybean meal	20
Fish meal	4
Wheat bran	4
Soybean oil	3.5
Rice bran	4
Premix	4
Total	100
Calculated nutrient content	
Dry matter (%)	87.87
Crude protein (%)	17.70
Ether extract (%)	7.10
Crude fiber (%)	3.12
Calcium (%)	0.70
Available phosphorus (%)	0.36
Digestible energy (MJ/kg)	14.26

The Premix provided the following nutrients per kilogram of diet: 2.0 g L-lysine HCl, 4.38 g calcium, 1.59 g phosphorus, 1.38 g Na, 2.07 g Cl, 90 mg Fe, 10 mg Cu, 10 mg Mn, 100 mg Zn, 0.15 mg I, 0.3 mg Se, 11,000 IU vitamin A, 2,000 IU vitamin D3, 40 IU vitamin E, 2 mg menadione, 3 mg thiamin, 6 mg riboflavin, 30 mg niacin, 20 mg pantothenic acid, 5 mg pyridoxine, 0.15 mg biotin, 1 mg folic acid, 40 μg vitamin B12, and 1.2 g choline.

Table S3. Composition of diets for LW weaned piglets

Ingredient	Content (%)
Corn	60
Soybean meal	21.5
Fish meal	5
Wheat bran	5
Soybean oil	3.5
Glucose	1
Premix	4
Total	100
Calculated nutrient content	
Dry matter (%)	87.87
Crude protein (%)	18.46
Ether extract (%)	6.62
Crude fiber (%)	2.99
Calcium (%)	0.72
Available phosphorus (%)	0.36
Digestible energy (MJ/kg)	14.25

The Premix provided the following nutrients per kilogram of diet: 1.0 g L-lysine HCl, 4.08 g calcium, 1.37 g phosphorus, 1.2 g Na, 1.8 g Cl, 90 mg Fe, 10 mg Cu, 10 mg Mn, 95 mg Zn, 0.15 mg I, 0.3 mg Se, 12,000 IU vitamin A, 1,800 IU vitamin D3, 45 IU vitamin E, 2 mg menadione, 3 mg thiamin, 5 mg riboflavin, 30 mg niacin, 20 mg pantothenic acid, 5 mg pyridoxine, 0.15 mg biotin, 1 mg folic acid, 35 μg vitamin B12, and 1.0 g choline.

Table S4. Composition of diets for SZL weaned piglets

Ingredient	Content (%)
Corn	62.8
Soybean meal	21
Fish meal	5
Wheat bran	4
Soybean oil	3.2
Premix	4
Total	100
Calculated nutrient content	
Dry matter (%)	87.77
Crude protein (%)	18.32
Ether extract (%)	6.38
Crude fiber (%)	2.97
Calcium (%)	0.72
Available phosphorus (%)	0.36
Digestible energy (MJ/kg)	14.23

The Premix provided the following nutrients per kilogram of diet: 1.0 g L-lysine HCl, 4.08 g calcium, 1.37 g phosphorus, 1.2 g Na, 1.8 g Cl, 90 mg Fe, 10 mg Cu, 15 mg Mn, 100 mg Zn, 0.15 mg I, 0.3 mg Se, 11,000 IU vitamin A, 1,800 IU vitamin D3, 40 IU vitamin E, 2 mg menadione, 3 mg thiamin, 6 mg riboflavin, 30 mg niacin, 20 mg pantothenic acid, 4 mg pyridoxine, 0.15 mg biotin, 1 mg folic acid, 40 µg vitamin B12, and 1.0 g choline.

Table S5. Composition of diets for CM weaned piglets

Ingredient	Content (%)
Corn	60
Soybean meal	26.5
Wheat bran	3
Soybean oil	3.5
Rice bran	3
Premix	4
Total	100
Calculated nutrient content	
Dry matter (%)	87.76
Crude protein (%)	17.57
Ether extract (%)	6.68
Crude fiber (%)	3.35
Calcium (%)	0.73
Available phosphorus (%)	0.37
Digestible energy (MJ/kg)	14.36

The Premix provided the following nutrients per kilogram of diet: 2.0 g L-lysine HCl, 6.24 g calcium, 2.73 g phosphorus, 1.78 g Na, 2.66 g Cl, 95 mg Fe, 10 mg Cu, 20 mg Mn, 95 mg Zn, 0.15 mg I, 0.3 mg Se, 12,000 IU vitamin A, 1,500 IU vitamin D3, 50 IU vitamin E, 2 mg menadione, 3 mg thiamin, 5 mg riboflavin, 30 mg niacin, 20 mg pantothenic acid, 5 mg pyridoxine, 0.15 mg biotin, 1 mg folic acid, 40 μg vitamin B12, and 1.0 g choline.

Table S6. Composition of diets for HM weaned piglets

Ingredient	Content (%)
Corn	59
Soybean meal	22.5
Fish meal	3
Wheat bran	3
Soybean oil	3.5
Rice bran	5
Premix	4
Total	100
Calculated nutrient content	
Dry matter (%)	87.91
Crude protein (%)	18.02
Ether extract (%)	7.13
Crude fiber (%)	3.23
Calcium (%)	0.77
Available phosphorus (%)	0.36
Digestible energy (MJ/kg)	14.3

The Premix provided the following nutrients per kilogram of diet: 2.0 g L-lysine HCl, 5.44 g calcium, 1.82 g phosphorus, 1.38 g Na, 2.07 g Cl, 90 mg Fe, 10 mg Cu, 15 mg Mn, 100 mg Zn, 0.15 mg I, 0.3 mg Se, 9,000 IU vitamin A, 1,600 IU vitamin D3, 40 IU vitamin E, 2 mg menadione, 3 mg thiamin, 6 mg riboflavin, 35 mg niacin, 20 mg pantothenic acid, 5 mg pyridoxine, 0.15 mg biotin, 1 mg folic acid, 40 μg vitamin B12, and 1.2 g choline.

Table S7. Composition of diets for NX weaned piglets

Ingredient	Content (%)
Corn	58
Soybean meal	22
Fish meal	4
Wheat bran	8
Soybean oil	4
Premix	4
Total	100
Calculated nutrient content	
Dry matter (%)	87.88
Crude protein (%)	18.38
Ether extract (%)	7.08
Crude fiber (%)	3.18
Calcium (%)	0.71
Available phosphorus (%)	0.37
Digestible energy (MJ/kg)	14.24

The Premix provided the following nutrients per kilogram of diet: 2.0 g L-lysine HCl, 4.38 g calcium, 1.59 g phosphorus, 1.38 g Na, 2.07 g Cl, 95 mg Fe, 8 mg Cu, 18 mg Mn, 95 mg Zn, 0.15 mg I, 0.3 mg Se, 11,000 IU vitamin A, 1,800 IU vitamin D3, 40 IU vitamin E, 2 mg menadione, 3 mg thiamin, 6 mg riboflavin, 35 mg niacin, 20 mg pantothenic acid, 5 mg pyridoxine, 0.15 mg biotin, 1 mg folic acid, 45 μg vitamin B12, and 1.0 g choline.

Table S8. Composition of diets for DLY finishing pigs

Ingredient	Content (%)
Corn	67
Soybean meal	20
Wheat bran	7
Soybean oil	2
Premix	4
Total	100
Calculated nutrient content	
Dry matter (%)	87.29
Crude protein (%)	15.32
Ether extract (%)	5.03
Crude fiber (%)	3.20
Calcium (%)	0.60
Available phosphorus (%)	0.19
Digestible energy (MJ/kg)	13.86

The Premix provided the following nutrients per kilogram of diet: 1.2 g L-lysine HCl, 5.17 g calcium, 1.03 g phosphorus, 0.99 g Na, 1.48 g Cl, 50 mg Fe, 5 mg Cu, 8 mg Mn, 50 mg Zn, 0.15 mg I, 0.2 mg Se, 5,000 IU vitamin A, 900 IU vitamin D3, 20 IU vitamin E, 1 mg menadione, 2 mg thiamin, 5 mg riboflavin, 20 mg niacin, 15 mg pantothenic acid, 2 mg pyridoxine, 0.15 mg biotin, 1 mg folic acid, 15 µg vitamin B12, and 0.8 g choline.

Table S9. Composition of diets for TM finishing pigs

Ingredient	Content (%)
Corn	55
Soybean meal	7
Wheat bran	8
Soybean oil	6
Alfalfa meal	20
Premix	4
Total	100
Calculated nutrient content	
Dry matter (%)	87.63
Crude protein (%)	12.08
Ether extract (%)	8.83
Crude fiber (%)	7.34
Calcium (%)	0.49
Available phosphorus (%)	0.22
Digestible energy (MJ/kg)	13.04

The Premix provided the following nutrients per kilogram of diet: 1.48 g calcium, 1.14 g phosphorus, 0.59 g Na, 0.89 g Cl, 60 mg Fe, 5 mg Cu, 5 mg Mn, 60 mg Zn, 0.2 mg I, 0.25 mg Se, 5,500 IU vitamin A, 1,000 IU vitamin D3, 20 IU vitamin E, 1 mg menadione, 2 mg thiamin, 4 mg riboflavin, 15 mg niacin, 15 mg pantothenic acid, 2 mg pyridoxine, 0.15 mg biotin, 1 mg folic acid, 15 μg vitamin B12, and 0.8 g choline.

Table S10. Composition of diets for LW finishing pigs

Ingredient	Content (%)
Corn	56
Soybean meal	16
Wheat bran	22
Soybean oil	2
Premix	4
Total	100
Calculated nutrient content	
Dry matter (%)	87.32
Crude protein (%)	14.70
Ether extract (%)	5.16
Crude fiber (%)	3.73
Calcium (%)	0.49
Available phosphorus (%)	0.17
Digestible energy (MJ/kg)	13.09

The Premix provided the following nutrients per kilogram of diet: 4.05 g calcium, 0.46 g phosphorus, 0.79 g Na, 1.18 g Cl, 60 mg Fe, 5 mg Cu, 6 mg Mn, 60 mg Zn, 0.2 mg I, 0.25 mg Se, 6,000 IU vitamin A, 900 IU vitamin D3, 20 IU vitamin E, 1 mg menadione, 2 mg thiamin, 3.5 mg riboflavin, 15 mg niacin, 15 mg pantothenic acid, 2 mg pyridoxine, 0.15 mg biotin, 1 mg folic acid, 12 µg vitamin B12, and 0.8 g choline.

Table S11. Composition of diets for SZL finishing pigs

Ingredient	Content (%)
Corn	70
Soybean meal	12
Wheat bran	10
Rice bran	4
Premix	4
Total	100
Calculated nutrient content	
Dry matter (%)	86.98
Crude protein (%)	12.91
Ether extract (%)	3.77
Crude fiber (%)	3.27
Calcium (%)	0.52
Available phosphorus (%)	0.22
Digestible energy (MJ/kg)	13.18

The Premix provided the following nutrients per kilogram of diet: 4.55 g calcium, 1.14 g phosphorus, 0.99 g Na, 1.48 g Cl, 65 mg Fe, 5 mg Cu, 6 mg Mn, 60 mg Zn, 0.2 mg I, 0.25 mg Se, 5,000 IU vitamin A, 800 IU vitamin D3, 25 IU vitamin E, 1 mg menadione, 2 mg thiamin, 4 mg riboflavin, 18 mg niacin, 15 mg pantothenic acid, 2 mg pyridoxine, 0.15 mg biotin, 1 mg folic acid, 12 μg vitamin B12, and 0.8 g choline.

Table S12. Composition of diets for CM finishing pigs

Ingredient	Content (%)
Corn	53
Soybean meal	11
Wheat bran	18
Soybean oil	2
Rice bran	12
Premix	4
Total	100
Calculated nutrient content	
Dry matter (%)	87.61
Crude protein (%)	13.42
Ether extract (%)	6.66
Crude fiber (%)	3.91
Calcium (%)	0.48
Available phosphorus (%)	0.17
Digestible energy (MJ/kg)	13.09

The Premix provided the following nutrients per kilogram of diet: 4.14 g calcium, 0.23 g phosphorus, 0.79 g Na, 1.18 g Cl, 60 mg Fe, 6 mg Cu, 5 mg Mn, 60 mg Zn, 0.2 mg I, 0.25 mg Se, 6,000 IU vitamin A, 900 IU vitamin D3, 20 IU vitamin E, 1 mg menadione, 2 mg thiamin, 3 mg riboflavin, 15 mg niacin, 15 mg pantothenic acid, 2 mg pyridoxine, 0.15 mg biotin, 1 mg folic acid, 12 μg vitamin B12, and 0.8 g choline.

Table S13. Composition of diets for HM finishing pigs

Ingredient	Content (%)
Corn	59
Soybean meal	13
Wheat bran	12
Soybean oil	3
Alfalfa meal	4
Rice bran	5
Premix	4
Total	100
Calculated nutrient content	
Dry matter (%)	87.50
Crude protein (%)	13.60
Ether extract (%)	6.67
Crude fiber (%)	4.30
Calcium (%)	0.50
Available phosphorus (%)	0.16
Digestible energy (MJ/kg)	13.40

The Premix provided the following nutrients per kilogram of diet: 3.67 g calcium, 0.46 g phosphorus, 0.79 g Na, 1.18 g Cl, 55 mg Fe, 5 mg Cu, 6 mg Mn, 60 mg Zn, 0.2 mg I, 0.25 mg Se, 5,000 IU vitamin A, 800 IU vitamin D3, 20 IU vitamin E, 1 mg menadione, 2 mg thiamin, 3 mg riboflavin, 15 mg niacin, 15 mg pantothenic acid, 2 mg pyridoxine, 0.2 mg biotin, 1 mg folic acid, 10 μg vitamin B12, and 0.8 g choline.

Table S14. Composition of diets for NX finishing pigs

Ingredient	Content (%)
Corn	66
Soybean meal	15
Wheat bran	8
Soybean oil	2
Rice bran	5
Premix	4
Total	100
Calculated nutrient content	
Dry matter (%)	87.35
Crude protein (%)	13.78
Ether extract (%)	5.72
Crude fiber (%)	3.29
Calcium (%)	0.55
Available phosphorus (%)	0.17
Digestible energy (MJ/kg)	13.71

The Premix provided the following nutrients per kilogram of diet: 4.73 g calcium, 0.68 g phosphorus, 0.79 g Na, 1.18 g Cl, 60 mg Fe, 5.5 mg Cu, 5 mg Mn, 60 mg Zn, 0.2 mg I, 0.25 mg Se, 5,500 IU vitamin A, 900 IU vitamin D3, 20 IU vitamin E, 1 mg menadione, 2 mg thiamin, 4 mg riboflavin, 14 mg niacin, 12 mg pantothenic acid, 2 mg pyridoxine, 0.15 mg biotin, 1 mg folic acid, 12 μg vitamin B12, and 0.8 g choline.
